# Niche creation improves bioaugmentation of an organic micropollutant degrader in oligotrophic waters

**DOI:** 10.1093/ismejo/wraf140

**Published:** 2025-07-04

**Authors:** Jinsong Wang, Bart Raes, Cato Debrabandere, Veerle van Aken, Sebastián Jaramillo-Toro, Steffen Waldherr, Benjamin Horemans, Dirk Springael

**Affiliations:** Division of Soil and Water Management, Department of Earth and Environmental Sciences, KU Leuven, Heverlee B-3001, Belgium; Department of Biotechnology, Delft University of Technology, 2629 HZ, Delft, The Netherlands; UNLOCK, Wageningen University & Research / Delft University of Technology, 6708 PB, Wageningen, The Netherlands; Division of Soil and Water Management, Department of Earth and Environmental Sciences, KU Leuven, Heverlee B-3001, Belgium; Division of Soil and Water Management, Department of Earth and Environmental Sciences, KU Leuven, Heverlee B-3001, Belgium; Division of Soil and Water Management, Department of Earth and Environmental Sciences, KU Leuven, Heverlee B-3001, Belgium; Division of Soil and Water Management, Department of Earth and Environmental Sciences, KU Leuven, Heverlee B-3001, Belgium; Chemical Reactor Engineering and Safety (CREaS), Department of Chemical Engineering, KU Leuven, Heverlee B-3001, Belgium; Department of Functional and Evolutionary Ecology, University of Vienna, 1030 Vienna, Austria; Division of Soil and Water Management, Department of Earth and Environmental Sciences, KU Leuven, Heverlee B-3001, Belgium; Chemical Reactor Engineering and Safety (CREaS), Department of Chemical Engineering, KU Leuven, Heverlee B-3001, Belgium; Division of Soil and Water Management, Department of Earth and Environmental Sciences, KU Leuven, Heverlee B-3001, Belgium

**Keywords:** micropollutant biodegradation, exploitative competition, microbial invasion, selective carbon source, bioaugmentation, niche creation, drinking water treatment

## Abstract

Bioaugmentation of sand filters is an alternative process for eliminating organic micropollutants in drinking water treatment. Bioaugmentation resembles an invasion process and niche availability is a prime determinant for successful invasion. This is particularly relevant for bioaugmentation of oligotrophic environments where organic micropollutants (OMPs) hardly provide a selective C-source and exploitative competition for the scarce intrinsic organic carbon exists between inoculated OMP-degraders and resident microbiota. Building on microbial invasion theories, we tested the hypothesis that the success of bioaugmentation and associated OMP degradation can be enhanced through niche creation by supplying a selective carbon source for the introduced degrader. Sand filter microbiota reduced growth of the 2,6-dichlorobenzamide degrading strain *Aminobacter niigataensis* MSH1 and 2,6-dichlorobenzamide degradation in different natural waters. This was counteracted by adding benzamide as a selective C-source for MSH1 resulting in a 3-fold faster 2,6-dichlorobenzamide biodegradation and a 6-fold increase in MSH1 growth. An additive biokinetic model underpredicted growth of MSH1 in the presence of sand filter microbiota suggesting that the community, despite its overall negative effect, supported MSH1 growth. Moreover, benzamide retarded 2,6-dichlorobenzamide degradation likely due to enzyme competitive inhibition. The results demonstrate the use of deliberately creating dedicated niches selective for the inoculum and the successful translation of ecological invasion theories into microbial community management, for improved bioaugmentation of complex communities.

## Graphical Abstract

**Graphical Abstract:**
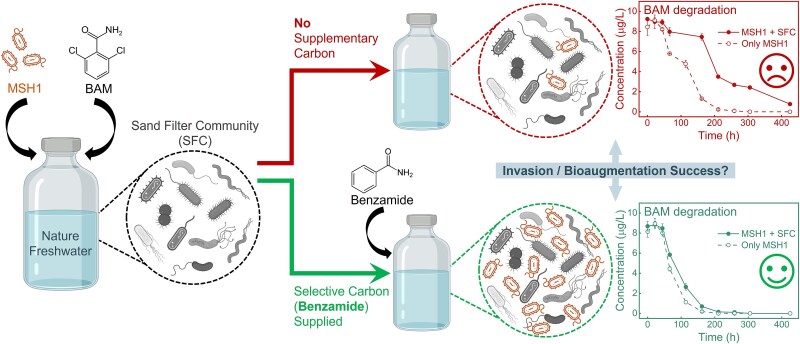

## Introduction

Microbial invasion is a fundamental process in microbial ecology [1–4]. A previously proposed framework defines invasion as the establishment of a foreign microbial species within a resident microbial community omitting the requirement that the invader negatively impacts the community [2]. In environmental biotechnology, dedicated microorganisms are introduced to improve removal of a target pollutant by biodegradation, a bioremediation effort referred to as “bioaugmentation” [5, 6]. Hence, bioaugmentation strongly resembles or can be considered as an invasion process aiming at the establishment of a deliberately introduced beneficial organism and should follow rules and experience bottle necks that are similar to invasion [2]. The diversity of the resident microbial community is proposed as a determining factor for the success of microbial invasion [7, 8]. Overall, invasion success decreases with increasing resident community diversity due to the exploitative competition between residents and invaders [2, 9–11]. Since distinct niches are occupied by diverse resident individuals with specialized resource utilization capabilities, resources available for invaders will become increasingly limited when more types of residents are present [2, 12, 13]. This implies that the success of establishing a deliberately introduced organism in an existing community, as in bioaugmentation, can be improved by antagonizing resource competition between residents and the introduced organism.

A feasible approach to reduce the strength of resource competition is to create a niche for the introduced organism [14]. A niche opportunity for foreign species to establish into a residence community might be provided by the high availability of resources on which that organism depends [14]. In microbial community assembly, the availability of specific carbon/energy sources determining growth kinetics and yields, are key in niche availability and establishment [9, 15]. Compared to a foreign organism, resident microbes are likely better adapted to use carbon sources intrinsic to their native environment, providing them a higher fitness to outcompete potential invaders [16–19]. Therefore, founding on ecological microbial invasion theories, it can be hypothesized that the deliberate addition of a C-source unique for the introduced organism and hence for which no competition exists with the indigenous community, provides that organism with its own niche and improves its establishment.

2,6-dichlorobenzamide (BAM) is a frequently detected organic micropollutant (OMP) in European groundwaters, causing concern for drinking water treatment plants (DWTPs) [20, 21]. Microbial degradation of OMPs involving bioaugmentation of sand filters exploited in DWTPs with specialized OMP catabolic bacteria, has been proposed as a cost-effective and sustainable alternative for OMP removal compared to physico-chemical treatment [22–24]. Recent studies showed improved BAM degradation in rapid sand filters bioaugmented with the BAM catabolic bacterium *Aminobacter niigataensis* MSH1 [25, 26]. However, long-term bioaugmentation effects were hampered by deteriorating MSH1 cell densities and activity likely due to shortage of energy carriers and competition for resources with endogenous microbiota in the highly oligotrophic environment of DWTPs [25, 26]. Clearly, bioaugmentation strategies need to be further improved.

In this study, we explore the hypothesis that niche creation by adding a C-source selective for a deliberately introduced OMP degrader, assists the establishment of that organism in a complex community and thus enhances OMP degradation in oligotrophic drinking water sources in the context of drinking water treatment. As a proof of principle and as a relevant model, *A. niigataensis* MSH1 was used as the inoculum organism and filter communities (SFCs) as native residents. We first investigated how SFCs and intrinsic organic carbon affected MSH1 growth and concomitant BAM degradation in natural freshwaters. We then tested whether addition of an exogenous carbon source, namely benzamide, that is selective for MSH1, introduced a competitive advantage and hence a niche for MSH1 while improving BAM degradation in the presence of SFCs. MSH1 is expected to use benzamide as a carbon source [27] while the isolation of benzamide utilising organisms requests extensive enrichment suggesting that benzamide catabolism is a rather rare metabolism in microbial communities [28]. MSH1’s expected ability to use benzamide as a C-source relies on the observation that the BbdA amidase responsible for conversion of BAM to 2,6-dichlorobenzoic acid, also converts benzamide into benzoic acid [27] and the prediction of a chromosomally encoded benzoic acid pathway in its genome sequence [29]. BbdA is a rather rare enzyme [30], further leveraging the selection of benzamide as the selective carbon source for MSH1. The experimental results were further interpreted by applying a recently reported additive biokinetic modelling framework adapted from Monod kinetics [31, 32]. This modelling framework predicts growth-linked OMP biodegradation at trace OMP concentrations in the presence of intrinsic assimilable organic carbon (AOC) and indigenous microorganisms that compete for this AOC with the OMP degrading inoculum, based on parameters estimated from experiments performed with individual actors, i.e. individual microbiota (in this study either MSH1 or the SFC) and individual C-sources. The modelling framework is though based solely on Monod kinetics and does not capture mechanisms like catabolite repression or more complex interactions between the inoculum and the indigenous microbes such as cross-feeding or interference competition. Hence, discrepancies between the experimental data and the model predictions, will indicate the occurrence of processes beyond classical Monod kinetics.

## Materials and methods

### Bacterial strains, sand filter communities, and chemicals

Two green fluorescent protein-labeled variants of *A. niigataensis* MSH1, namely the previously reported MSH1-*gfp* [33] and the recently constructed MSH1-*mClover*, were used (see details in Text S1). Both labelled variants show growth kinetics on BAM that are not statistically different from each other or from the wild type (Fig. S1). Indigenous SFCs were extracted from three sand filters exploited in three different DWTPs located in either Haacht, Sinaai, and Kluizen in Belgium, kindly provided by De Watergroep and referred to as SFC-HA, SFC-SI, and SFC-KL, respectively. Details on SFC extraction from the sand filter material are provided in Text S2. BAM, glucose, and benzamide were purchased from Sigma-Aldrich (Belgium).

### Preparation of AOC-restricted media and glass/plastic ware

To reduce background growth on contaminating AOC (originating from sources beyond the used waters) and the consequent impacts on bioaugmentation and degradation of BAM at trace concentrations, all experiments were performed in AOC-restricted media and glass/plastic ware. AOC-restricting treatment was performed as described [34].

### Environmental waters

Four freshwaters were sampled at different locations in Belgium, including the Dijle river, a pond in Zoete Waters park, and two intake waters from sand filters operated at DWTPs located in Kluizen and Sinaai. The four waters were named water-DI, water-ZW, water-KL, and water-SI, respectively. Water samples were collected in AOC-restricted Schott bottles followed by sterilization and chemical characterization (see Text S3).

### Growth/biodegradation experiments

Experiments were performed in 40 ml borosilicate glass vials with caps containing silicone PTFE lined septa (Sigma Aldrich, Belgium) and containing 10 ml of environmental water treated as described above. Different inocula and carbon sources were added to the vials in four different experiments. In a first experiment, the effects of SFCs on the growth of MSH1 and concomitant BAM degradation were studied in different natural waters. Subsequently, we investigated whether benzamide functioned as a selective carbon source for MSH1 by comparing the growth of MSH1 and an SFC on benzamide. This was followed by an experiment exploring the effect of benzamide as an auxiliary C-source on MSH1 mediated BAM degradation. A final experiment explored whether addition of benzamide affected MSH1 growth and BAM degradation in the presence of SFC under diverse competition strengths. The experimental design, detailed in Text S4 and Table S1, always used a triplicate set-up incubated on a shaker (125 rpm) in the dark at 25°C with initial inoculum densities of 10^3^ cells/ml. In the first three experiments, MSH1-*gfp* was used. In the fourth and last experiment, MSH1-*mClover* was used.

### Analytical methods

Samples were collected regularly for quantifying cell densities and concentrations of BAM/benzamide. The GFP fluorescence of MSH1-*gfp* is not strong enough to allow detection by flow cytometry (FC) and hence MSH1-*gfp* cannot be directly recognized by FC when combined with an SFC. In case MSH1-*gfp* was used in combination with an SFC, total cell densities (SFC plus MSH1-*gfp*) were determined by FC whereas MSH1-*gfp* CFU density was measured by plating and scoring green fluorescent colonies as outlined in Text S5. No significant differences between CFU-based MSH1*-gfp* cell counts and FC-based MSH1-*gfp* cell counts were noted (*P* = .30, Fig. S2) and therefore, SFC cell densities were calculated by subtracting MSH1-*gfp* CFU densities from the total cell densities. In case MSH1-*gfp* was used without adding an SFC or in case only the SFC was added, their respective cell densities were directly determined by FC as previously reported [34]. In contrast to MSH1-*gfp*, the fluorescence of MSH1-*mClover* is strong enough for direct detection by FC. In case of mixed systems containing both MSH1-*mClover* and an SFC, total cell densities (SFC plus MSH1-*mClover*) and MSH1-*mClover* cell densities were determined by FC. SFC densities were subsequently calculated by subtracting MSH1-*mClover* cell densities from the total cell densities. BAM and benzamide concentrations were determined by Ultra High Performance Liquid Chromatography coupled with a LC-MS8045 triple quadrupole mass spectrometer (Shimadzu Corp, Japan). Details on all analyses are in Texts S5 and S6. AOC concentrations were determined using a previously reported standard protocol [35]. Glucose concentrations were not quantified.

### Model simulation of kinetic parameters for BAM biodegradation and MSH1 growth

To understand the effects of the selective carbon source (i.e. benzamide), coincidental carbon sources (i.e. AOC and glucose), and the indigenous microbial community (i.e. SFC) on MSH1 growth and BAM biodegradation, the data generated from the final experiment were analysed by applying a previously reported biokinetic modelling framework (Eqs. (1–7)) [31] and using a non-linear regression method. The model was slightly adapted by altering the equation of BAM utilization by MSH1 into a pseudo-first order kinetic equation [36] as BAM utilization by MSH1 occurs through non-growth linked biodegradation at the concentration of 10 μg/L used in this study [34]. First, individual kinetic parameters were estimated for each individual system partner (i.e. MSH1 and the SFC-SI) under conditions where only one of them was present, i.e. MSH1 growing on intrinsic AOC, glucose or benzamide, MSH1 degrading BAM, and SFC-SI growing on either intrinsic AOC or glucose, as detailed in Fig. S3. To this end, the concentrations of different C-sources were normalized into μg-C/L. The decay rate of MSH1 was estimated from the experimental data using the first-order decay model (Eq. 8) [37]. Table 1 shows all used model equations whereas Table S2 lists all the definitions and values of the kinetic parameters. The estimated parameter values were then applied to model simulations to predict growth and biodegradation dynamics in more complex conditions (e.g. co-presence of MSH1 and SFCs or co-addition of intrinsic AOC, glucose, and benzamide). No further parameter optimizations were performed for these simulations. The initial conditions, such as the starting cell density and substrate concentration, for the complex configurations were obtained from the experimental data. Deviations between the experimental data and the model output provided insights into mechanisms at play that cannot fully be explained by the individual kinetics. Model simulations were performed in Python (version 3.8) [38] and the differential equations (Eqs. (1–7)) were simultaneously solved with parameters sampled from their 95% confidence intervals. Model fitting performance was evaluated using relative mean absolute error (RMAE), percent bias (PBIAS), and cosine distance (as detailed in Text S7) [39].

**Table 1:** Overview of the equations used for model simulation. The definition of each kinetic parameter is listed in Table S2.

**Equation Name**	**Equation Formula**	**Equation Number**
MSH1 biomass growth	$\frac{\mathrm{d}{\mathrm{X}}_{\mathrm{m}}}{\mathrm{d}\mathrm{t}}={\mathrm{R}}_{\mathrm{m}\mathrm{AOC}}\frac{{\mathrm{S}}_{\mathrm{m}\mathrm{AOC}}{\mathrm{X}}_{\mathrm{m}}}{{\mathrm{K}}_{\mathrm{m}\mathrm{AOC}}+{\mathrm{S}}_{\mathrm{m}\mathrm{AOC}}}+{\mathrm{R}}_{\mathrm{Benz}}\frac{{\mathrm{S}}_{\mathrm{Benz}}{\mathrm{X}}_{\mathrm{m}}}{{\mathrm{K}}_{\mathrm{Benz}}+{\mathrm{S}}_{\mathrm{Benz}}}+{\mathrm{R}}_{\mathrm{m}\mathrm{Glc}}\frac{{\mathrm{S}}_{\mathrm{Glc}}{\mathrm{X}}_{\mathrm{m}}}{{\mathrm{K}}_{\mathrm{m}\mathrm{Glc}}+{\mathrm{S}}_{\mathrm{Glc}}}-{\mathrm{D}}_{\mathrm{m}}{\mathrm{X}}_{\mathrm{m}}$	(1)
SFC biomass growth	$\frac{\mathrm{d}{\mathrm{X}}_{\mathrm{s}}}{\mathrm{d}\mathrm{t}}={\mathrm{R}}_{\mathrm{s}\mathrm{AOC}}\frac{{\mathrm{S}}_{\mathrm{mAOC}}{\mathrm{X}}_{\mathrm{s}}}{{\mathrm{K}}_{\mathrm{s}\mathrm{AOC}}+{\mathrm{S}}_{\mathrm{mAOC}}}+{\mathrm{R}}_{\mathrm{s}\mathrm{AOC}}\frac{{\mathrm{S}}_{\mathrm{s}\mathrm{AOC}}{\mathrm{X}}_{\mathrm{s}}}{{\mathrm{K}}_{\mathrm{s}\mathrm{AOC}}+{\mathrm{S}}_{\mathrm{s}\mathrm{AOC}}}+{\mathrm{R}}_{\mathrm{s}\mathrm{Glc}}\frac{{\mathrm{S}}_{\mathrm{Glc}}{\mathrm{X}}_{\mathrm{s}}}{{\mathrm{K}}_{\mathrm{s}\mathrm{Glc}}+{\mathrm{S}}_{\mathrm{Glc}}}-{\mathrm{D}}_{\mathrm{s}}{\mathrm{X}}_{\mathrm{s}}$	(2)
BAM utilization	$\frac{{\mathrm{dS}}_{\mathrm{BAM}}}{\mathrm{dt}}=-{\mathrm{Q}}_{\mathrm{BAM}}{\mathrm{X}}_{\mathrm{m}}{\mathrm{S}}_{\mathrm{BAM}}$	(3)
AOC_MSH1_ utilization	$\frac{{\mathrm{dS}}_{\mathrm{m}\mathrm{AOC}}}{\mathrm{dt}}=\frac{-{\mathrm{R}}_{\mathrm{m}\mathrm{AOC}}}{{\mathrm{Y}}_{\mathrm{m}\mathrm{AOC}}}\frac{{\mathrm{S}}_{\mathrm{m}\mathrm{AOC}}{\mathrm{X}}_{\mathrm{m}}}{{\mathrm{K}}_{\mathrm{m}\mathrm{AOC}}+{\mathrm{S}}_{\mathrm{m}\mathrm{AOC}}}+\frac{-{\mathrm{R}}_{\mathrm{s}\mathrm{AOC}}}{{\mathrm{Y}}_{\mathrm{s}\mathrm{AOC}}}\frac{{\mathrm{S}}_{\mathrm{m}\mathrm{AOC}}{\mathrm{X}}_{\mathrm{s}}}{{\mathrm{K}}_{\mathrm{s}\mathrm{AOC}}+{\mathrm{S}}_{\mathrm{m}\mathrm{AOC}}}$	(4)
AOC_SFC_ utilization	$\frac{{\mathrm{dS}}_{\mathrm{s}\mathrm{AOC}}}{\mathrm{dt}}=\frac{-{\mathrm{R}}_{\mathrm{s}\mathrm{AOC}}}{{\mathrm{Y}}_{\mathrm{s}\mathrm{AOC}}}\frac{{\mathrm{S}}_{\mathrm{s}\mathrm{AOC}}{\mathrm{X}}_{\mathrm{s}}}{{\mathrm{K}}_{\mathrm{s}\mathrm{AOC}}+{\mathrm{S}}_{\mathrm{s}\mathrm{AOC}}}$	(5)
Benzamide utilization	$\frac{{\mathrm{dS}}_{\mathrm{Benz}}}{\mathrm{dt}}=\frac{-{\mathrm{R}}_{\mathrm{Benz}}}{{\mathrm{Y}}_{\mathrm{Benz}}}\frac{{\mathrm{S}}_{\mathrm{Benz}}{\mathrm{X}}_{\mathrm{m}}}{{\mathrm{K}}_{\mathrm{Benz}}+{\mathrm{S}}_{\mathrm{Benz}}}$	(6)
Glucose utilization	$\frac{{\mathrm{dS}}_{\mathrm{Glc}}}{\mathrm{dt}}=\frac{-{\mathrm{R}}_{\mathrm{m}\mathrm{Glc}}}{{\mathrm{Y}}_{\mathrm{m}\mathrm{Glc}}}\frac{{\mathrm{S}}_{\mathrm{Glc}}{\mathrm{X}}_{\mathrm{m}}}{{\mathrm{K}}_{\mathrm{m}\mathrm{Glc}}+{\mathrm{S}}_{\mathrm{Glc}}}+\frac{-{\mathrm{R}}_{\mathrm{s}\mathrm{Glc}}}{{\mathrm{Y}}_{\mathrm{s}\mathrm{Glc}}}\frac{{\mathrm{S}}_{\mathrm{Glc}}{\mathrm{X}}_{\mathrm{s}}}{{\mathrm{K}}_{\mathrm{s}\mathrm{Glc}}+{\mathrm{S}}_{\mathrm{Glc}}}$	(7)
MSH1 biomass decay^a^	$\mathrm{Ln}\frac{{\mathrm{X}}_{\mathrm{m},\mathrm{Final}}}{{\mathrm{X}}_{\mathrm{m},\operatorname{Max}}}=-{\mathrm{D}}_{\mathrm{m}}\mathrm{t}$	(8)

(**^a^:** the decay model only applied to MSH1, as no decay of SFC was observe in this study.)

## Results

### Chemical characteristics of the natural waters

The chemical characteristics of the four freshwaters used in this study are shown in Tables S3 and S4. pH values ranged between 7.5 and 8.5 implying that BAM is present in its undissociated form based on its pKa value (14.7). To sustain aerobic bacterial growth, a C:N:P ratio of 100:5:1 is often used as a rule of thumb [40]. This C:N:P ratio, based on TOC, was met for water-DI and water-ZW whereas phosphate appears limiting in water-KL and water-SI. However, AOC is typically only a small fraction of the TOC (0.1%–6%) [41] and likely the limiting nutrient in all waters, including water-KL and water-SI.

### Influence of sand filter community on MSH1 growth and BAM degradation

**Figure 1:**
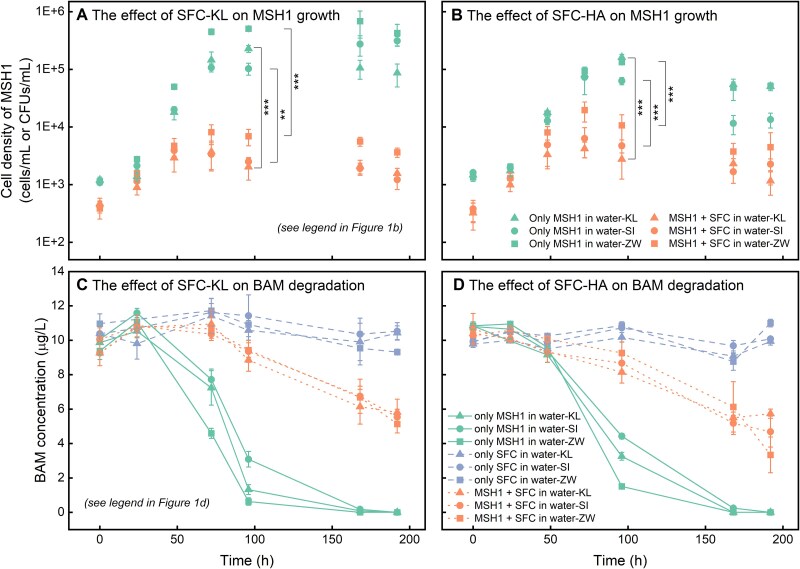
Effect of the presence of SFC-KL and SFC-HA on the growth of MSH1 (**A** and **B**) and on MSH1-mediated BAM degradation (**C** and **D**) in three different freshwaters (water-KL, water-ZW, and water-SI). In panels a and b, the unit of MSH1 cell density is cells/ml when only MSH1 was inoculated, whereas the unit is CFUs/ml when MSH1 and SFC were co-present. The statistical analysis indicated negligible differences between CFU and total cell units (Fig. S2). A *t*-test was used to analyse the significance of differences in MSH1 cell densities between groups, “^**^” means *P* value is .001–.01; “^***^” means *P* value is .0001–.001. Error bars represent standard deviation of three biological replicates.

The growth of MSH1 and concomitant BAM degradation were monitored in water-KL, water-ZW, and water-SI, in either the presence or the absence of one of two different SFCs, to understand how co-existing indigenous bacteria affect MSH1 growth and BAM degradation. SFC-KL and SFC-HA were used as SFC communities. In all three waters, both SFCs significantly suppressed the growth of MSH1 (*P* < .05) (Fig. 1A and B). Maximum MSH1 cell densities were around 75-fold (SFC-KL) and 19-fold (SFC-HA) lower in the presence of SFC than in the absence of the SFC (Fig. 1A and B). Apparently, AOC available for MSH1 (AOC_MSH1_) was significantly reduced in the presence of the SFCs (*P* < .05) (Fig. S4). In addition, the presence of the SFCs clearly affected BAM biodegradation. BAM became completely depleted in the absence of the SFCs whereas approximately 57.9 ± 6.1% (SFC-KL) and 43.9 ± 11.4% (SFC-HA) remained in the presence of the SFCs (Fig. 1C and D). In contrast, MSH1 did not affect SFC growth (Fig. S5) and AOC available for the SFC (AOC_SFC_) was similar whether or not MSH1 was present (*P* > .05) (Fig. S4). It was concluded that MSH1 has a competitive disadvantage for growth on AOC in the presence of co-incidental microbiota (i.e. SFCs) in freshwaters, leading to poor establishment and decreased BAM degradation rates compared to systems without indigenous microbiota.

### Benzamide is a selective carbon source for MSH1

The rare BbdA amidase that converts BAM to 2,6-DCBA in MSH1, also converts benzamide to benzoic acid [27]. Meanwhile MSH1 likely uses benzoate as a C-source since annotation of its genome sequence predicted the presence of a chromosomally encoded catechol *ortho*-cleavage pathway [27, 29]. Together, it implies that benzamide is a potential selective C-source for MSH1. To confirm, the growth rate and yield of MSH1 and SFC-SI on benzamide were examined in water-DI amended with either 100 μg/L and 600 μg/L benzamide. MSH1 showed clear growth on benzamide reaching higher final cell densities in benzamide-amended water-DI compared to the background growth in water-DI without benzamide (Fig. S6A). AOC_MSH1_ in water-DI was extremely low, i.e. 0.67 ± 0.02 μg-C/L, implying that the water’s intrinsic AOC contributed minimally to MSH1 growth and that growth was primarily linked with benzamide utilization. SFC-SI did not display growth on benzamide (Fig. S6B). Benzamide was completely consumed by MSH1 within 72 hours at both concentrations (Fig. S6C and D). Benzamide degradation was also observed for SFC-SI in water-DI amended with 600 μg/L benzamide (Fig. S6D) but only initiated after 216 hours and only 37.7 ± 1.3% of the benzamide was converted after 288 hours (Fig. S6D). It was concluded that benzamide is a C-source for MSH1 and selective for MSH1 against SFCs.

**Figure 2:**
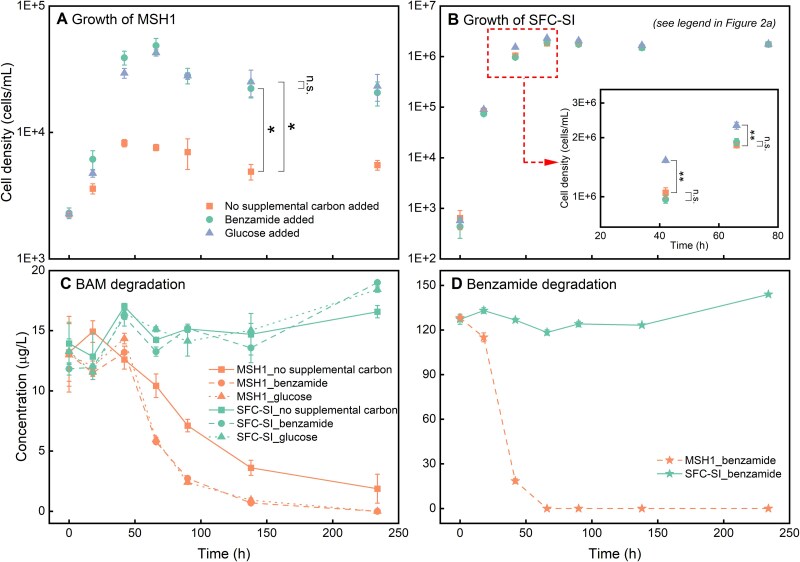
Effect of benzamide and glucose on growth of MSH1 and concomitant BAM biodegradation and on growth of SFC-SI. (**A**) Growth of MSH1, (**B**) growth of SFC-SI, (**C**) BAM biodegradation in the presence of either MSH1 or SFC-SI, (**D**) benzamide degradation in the presence of either MSH1 or SFC-SI. A *t*-test was used to analyse the significance of differences in SFC-SI cell densities between groups, “n.s.” indicates no significant difference with *P* > .05; “^*^” means *P* value is .01–.05; “^**^” means *P* value is .001–.01. Error bars represent standard deviation of three biological replicates.

It was subsequently explored whether the supplementation of benzamide enhances MSH1 growth and thereby accelerates BAM degradation, compared to a system without benzamide. MSH1 was inoculated in water-DI either without or with supplementing 130 μg/L (90 μg-C/L) benzamide. To compare, the growth of MSH1 in water-DI supplemented with 225 μg/L (90 μg-C/L) glucose was examined. BAM was always added at approximately 10 μg/L. Since minor benzamide degradation by SFC-SI was observed previously, SFC-SI was inoculated in a separate set of vials to assess its growth on glucose and benzamide. MSH1 final cell densities were significantly higher in systems with benzamide or glucose compared to systems without any extraneous carbon sources (*P* < .03, Fig. 2A). Similar growth rates and cell densities were reached with either benzamide or glucose (*P* = .53, Fig. 2A). Moreover, addition of glucose and benzamide increased the BAM degradation rate (Figs. 2C and S7) without significant difference between both C-sources (*P* = .4, Fig. S7D). MSH1 completely consumed benzamide within 66 hours (Fig. 2D). The addition of benzamide did not improve the growth of SFC-SI (*P* > .15) whereas the SFC-SI maximum cell density increased 26% when glucose was added (Fig. 2B). SFC-SI degraded neither benzamide (Fig. 2D) nor BAM (Fig. 2C). We conclude that benzamide (i) positively affects MSH1-mediated BAM degradation by increasing its cell densities and (ii) is hardly used as a C-source by the tested SFC. Therefore, benzamide appears a suitable selective carbon source for MSH1.

### Benzamide counteracts the negative effect of an SFC on MSH1-mediated BAM degradation

**Figure 3:**
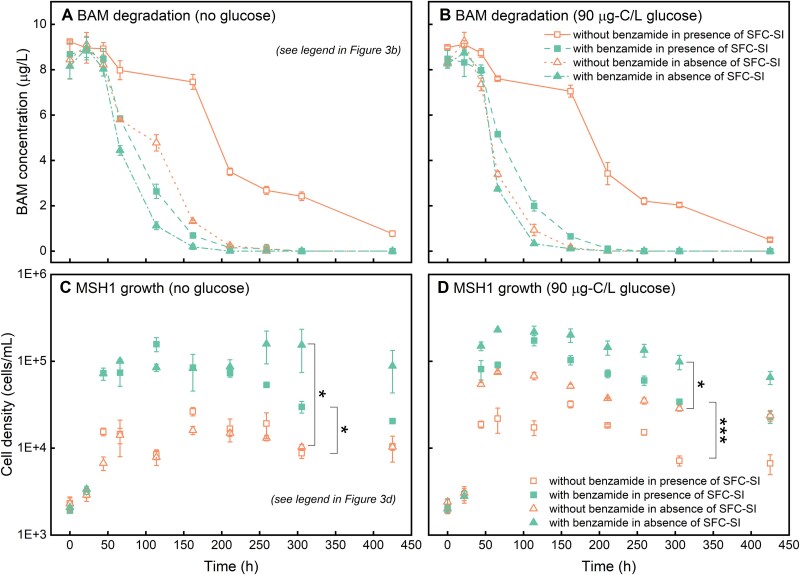
Effect of benzamide and glucose on growth of MSH1 and concomitant BAM biodegradation in the presence and absence of SFC-SI. (**A**) BAM degradation in systems without glucose, (**B**) BAM degradation in systems amended with 90 μg-C/L glucose, (**C**) growth of MSH1 in systems without glucose, (**D**) growth of MSH1 in systems amended with 90 μg-C/L glucose. A *t*-test was used to analyse the significance of differences in MSH1 cell densities between groups, “^*^” means *P* value is .01–.05; “^***^” means *P* value is .0001–.001. Error bars represent standard deviation of three biological replicates.

A final experiment examined whether the addition of benzamide counteracted the negative effect of SFCs on MSH1 growth and MSH1-mediated BAM degradation due to competition for AOC. Glucose, a C-source used by both MSH1 and the SFC, was added as additional AOC at four different concentrations (i.e. 0, 75, 225, and 500 μg/L) to initiate diverse competition strengths. Water-DI and SFC-SI were used as freshwater background and SFC, respectively. Addition of benzamide enhanced both BAM degradation and MSH1 growth, not only when SFC-SI was absent as shown above (Fig. 2) but also in the presence of SFC-SI (Fig. S8). Specifically, supplementing benzamide resulted in a threefold increase in BAM degradation rate (Figs. S8A, S9, and S10) and a sixfold improvement in MSH1 growth yield (Fig. S8C). Benzamide was consumed within 66 hours (Fig. S8B). SFC-SI neither degraded nor grew on benzamide (Fig. S8D). As observed previously, the presence of SFC-SI negatively affected MSH1 growth (Fig. 3C and D) and decreased BAM degradation rates (Figs. 3A and B, S11, and S12). BAM/benzamide biodegradation and MSH1 growth was independent from adding extra glucose (Figs. S8 and S13) and no obvious changes in SFC-SI yields were observed (Fig. S8D). Likely, most of the glucose was utilized by SFC-SI before being assimilated by MSH1, and none of the glucose concentrations (up to 500 μg/L (200 μg-C/L)) did significantly boost the SFC cell densities compared to the intrinsic AOC available for SFC-SI in water-DI (182.9 ± 23.2 μg-C/L) as determined before in the previous experiment. BAM degradation rates were almost completely restored upon benzamide addition (Fig. 3A and B), as no significant difference was observed in the kinetic constants of BAM biodegradation between the treatment that contained benzamide and SFC-SI and the control lacking them (*P* > .05) (Fig. S14C and F). Moreover, the addition of benzamide significantly enhanced MSH1 growth in both treatments with and without extra glucose (*P* < .04, Fig. 3C and D), despite the higher intrinsic AOC_SFC_ (182.9 ± 23.2 μg-C/L) compared to AOC_MSH1_ (0.67 ± 0.02 μg-C/L) in water-DI. The above results demonstrated that the competition for AOC can be counteracted by adding benzamide and hence that the supply of a unique C-source for the invader, i.e. MSH1 creates a niche for the invader and improves its establishment and functionality.

### Prediction of MSH1 growth/BAM biodegradation kinetics based on individual systems and comparison with experimental data in complex systems

**Figure 4:**
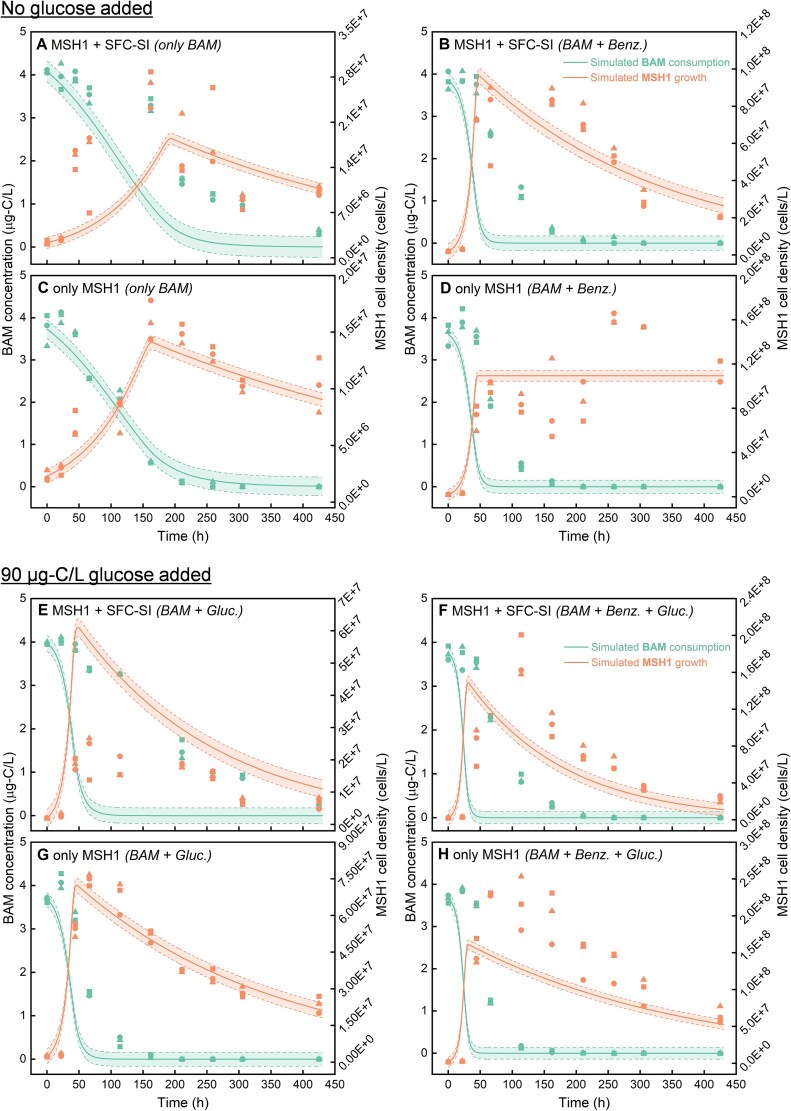
Comparison of simulated data (solid lines) by the biokinetic model framework with 95% confidence intervals (shadow areas) to the triplicate measured data (circle, triangle and square symbols) obtained from different test groups in the final experiment that explored the effect of auxiliary C-source on growth of MSH1 and concomitant BAM degradation in the presence of SFC-SI (see Table S1), including (**A**) group 1 (MSH1+ SFC-SI inoculated, only BAM added), (**B**) group 5 (MSH1+ SFC-SI inoculated, both BAM and benzamide added), (**C**) group 9 (only MSH1 inoculated, only BAM added), (**D**) group 11 (only MSH1 inoculated, both BAM and benzamide added), (**E**) group 3 (MSH1+ SFC-SI inoculated, both BAM and glucose added), (**F**) group 7 (MSH1+ SFC-SI inoculated, BAM, benzamide, and glucose added), (**G**) group10 (only MSH1 inoculated, both BAM and glucose added), (**H**) group 12 (only MSH1 inoculated, BAM, Benzamide, and glucose added). The simulated and experimental data of benzamide degradation and SFC-SI growth are present in Fig. S16 and Fig. S17, respectively. The overall results of model evaluation statistics are summarized in Table S5. “Benz.” refers to “benzamide” and “Gluc.” to “glucose”.

A biokinetic model (Table 1) was applied—based on kinetic parameters estimated from simpler configurations (such as only containing MSH1 or only BAM added) in a last experiment (Figs. S3 and S15) —to predict MSH1/SFC-SI growth and BAM/benzamide degradation in the more complex system configurations (Fig. 4). This way, potential discrepancies between the observed and predicted dynamics of growth and BAM utilisation are revealed, indicating potential processes beyond Monod kinetics that determine the outcome of the bioaugmentation effort. The model predictions of MSH1 growth and BAM degradation are presented in Fig. 4 together with the experimental data whereas the simulation results of benzamide biodegradation and SFC growth are shown in Figs. S16 and S17, respectively. The predictions are largely based on Monod kinetics without interactions between different actors for instance between SFC-SI and MSH1 or effects of one substrate on another. In case the experimental data fit with the model, it means that such interactions do not exist and all kinetics are explained by Monod kinetics. That was not the case, as evidenced by the statistical assessment of model fitting performance presented in Table S5. For the conditions without glucose (lower background AOC concentrations hence less competition between MSH1 and SFC-SI) (Fig. 4A–D), the model predicted well the growth of MSH1 (RMAE<30%, PBIAS<10%) (Table S5) except for the condition that contained SFC-SI without addition of benzamide (Fig. 4A), where BAM degradation was overestimated (PBIAS = 32.6%) whereas the growth of MSH1 was underpredicted (PBIAS = 24.3%) (Table S5). This indicates that the presence of SFC-SI negatively affected BAM degradation, while MSH1 experienced some positive interactions with SFC-SI for growth. When benzamide was present, the model overestimated BAM biodegradation even in the absence of SFC-SI (PBIAS = 46.5%) (Table S5 and Fig. 4D), suggesting that benzamide negatively affected BAM degradation compared to the condition without benzamide (Fig. 4C). For the conditions in which glucose was added (higher background AOC concentrations hence more competition between MSH1 and SFC), the model overestimated the growth of SFC-SI (Fig. S17E and F) (PBIAS = −24.9% and −17.3%, Table S5) and BAM biodegradation (Fig. 4E–H) (PBIAS > 40%, Table S5). The model also overpredicted MSH1 growth in the presence of SFC-SI when glucose was added (Fig. 4E) (PBIAS = −135.3%, Table S5) which is in contrast with the condition without glucose (Fig. 4A). This might be explained by a stronger competition between MSH1 and SFC-SI when glucose is present hence negatively affecting MSH1 growth. In addition, BAM degradation was overpredicted in the presence of glucose when only MSH1 was inoculated (Fig. 4G) (PBIAS = 40.4%, Table S5), indicating that the presence of glucose negatively influenced BAM degradation. Overall, benzamide counteracted the exploitative competition by SFC-SI by improving MSH1 growth and BAM degradation; however, it retarded BAM degradation compared to conditions without benzamide.

## Discussion

Our results demonstrate that the presence of an indigenous community negatively affects the growth of MSH1 and associated BAM biodegradation likely due to the exploitative competition. The results are reminiscent to observations reported in a similar study showing that the presence of a freshwater community diminished growth of the carbofuran catabolic *Novosphingobium* sp. KN65.2 and concomitant carbofuran degradation compared to systems without the community in natural waters [32]. The authors attributed the decreased growth and carbofuran degradation to either exploitative competition for the AOC between degrader and community members [32] and alternatively that intermediates of carbofuran catabolism released by KN65.2 were channelled into consumption by community members instead of being consumed by KN65.2 [32]. The latter though is unlikely in the current study as the extremely low BAM concentrations (10 μg/L) represented only a minor fraction of the total available organic carbon meaning that any released metabolites could only be present at trace concentrations. In contrast, in that previously reported study [32], carbofuran was present at a 100-fold higher concentration (1 mg/L) and hence its degradation might have led to relatively high concentrations of metabolites. The supposed exploitative competition by the SFCs might be due to direct competition for the AOC itself or for other nutrients that are used by the SFC to degrade the AOC. Often exploitative competition increases with an increasing community diversity since more species will occupy more niches [1, 7, 8]. Although we did not determine the SFC community diversity in this study, previous studies showed that DWTP sand filters contain highly diverse communities including the sand filters of the Kluizen and Sinaii DWTPs [42–44]. Moreover, it is plausible to assume that DWTP sand filter bacteria are more adapted to nutrient-poor freshwater environments compared to MSH1 that was isolated under high nutrient conditions from soil and hence that they use the scarce nutrients in the water more efficiently [11, 45]. This observation suggests that competition for intrinsic organic carbon indeed plays an important role in the bioaugmentation process, specifically, in the establishment of a niche for an inoculated OMP degrader like MSH1, and hence will impact bioaugmentation efforts in DWTP systems for OMP removal. Previously, the availability of intrinsic energy carriers was suggested as a crucial determinant for bioaugmentation with MSH1 in DWTP sand filters both in laboratory- and pilot-scale systems while exploitative competition was shown to occur between SFC isolates and MSH1 [25, 26, 33, 46]. We show here that MSH1 might only use a small fraction of the AOC available for microbial consumption in freshwaters and that there is a fierce competition for that AOC or the nutrients required for AOC assimilation when resident SFC organisms are present. This competition might further depend on different factors such as the quantity and quality of the organic carbon available [11, 32]. Moreover, the type of SFC seems to play a role, since addition of two different SFCs in this study to the same water resulted into different effects on the MSH1 population and the available AOC_MSH1_, likely linked with differences in composition of the different SFCs.

The predictive modelling results suggested that additional mechanisms beyond exploitative competition play a role in MSH1 establishment since it underestimated the growth of MSH1 in the presence of SFC under the condition without supplemental C-source. This might indicate that, despite the negative effect of the SFC on overall MSH1 growth and BAM degradation, MSH1 was also profiting from its presence and hence that positive interactions existed from the SFC towards MSH1. This can be for instance explained by the exchange of carbon or other growth factors from the SFC to MSH1, i.e. AOC available for the SFC was converted into AOC available for MSH1. Similar observations were reported previously [32] SFCs have been shown to contain bacteria that cooperate with MSH1 and support its fitness [46]. Although growth of MSH1 was underestimated by the modelling, degradation of BAM was overestimated, suggesting decoupling of BAM degradation and MSH1 cell numbers. A plausible explanation would be that exchanged carbon substrates from the SFC populations result into catabolite repression. Even though catabolic repression of BAM degradation in MSH1 was not reported before, catabolic repression has been observed for other aromatic catabolic pathways [47–50]. Moreover, the positive effects disappeared when glucose was added. The addition of glucose might have promoted the growth and fitness of certain SFC populations which outcompeted or were disadvantageous for the bacteria that interacted positively with MSH1 for instance by means of higher order interactions [51]. Several previous studies suggest that pairwise interactions between residents and/or higher order interactions can impact invasion including invasion of SFCs by MSH1 [52–54]. Characterization of the microbial community structure of different SFCs and their dynamics in systems with and without MSH1 as well as of the chemical composition of AOC, should enable deeper insight in the SFC populations and mechanisms underlying the exploitative competition and other interactions governing bioaugmentation.

Benzamide was selected and experimentally appointed as a C-source to create a niche and avert exploitative competition by indigenous microbiota in support of MSH1 growth and BAM degradation. A major requirement for such a C-source is that it is quite unique for the introduced degrader and is not used by residents. That was indeed the case. Competition from the tested SFC for the use of benzamide by MSH1 was not observed. Although some degradation was observed by SFC-SI in water-DI after 200 hours when applying a higher concentration of benzamide (600 μg/L), no growth was observed. Benzamide is the non-chlorinated variant of BAM and its first catabolic step in MSH1 uses the same amidase enzyme BbdA that is used for BAM catabolism [27, 29]. The BbdA phenotype appears quite rare in environmental microbial communities including communities of DWTP sand filters [30] but alternative benzamide amidases have been reported in various benzamide catabolic bacteria belonging to different genera including Gram-negative and Gram-positive bacteria [28, 55, 56]. These benzamide amidases belong to the amidase signature family, have only a low amino acid similarity with BbdA of MSH1 [27] and they show a 20 to 200 higher Km hence lower affinity for benzamide compared to BbdA of MSH1 [27]. Hence, they are less suitable for degradation of benzamide at the trace concentrations (μg/L range) used in this study while delivering a competitive advantage for MSH1 expressing the BbdA high affinity amidase. However, since the targeted communities (i.e. SFC) used in our study are from oligotrophic environments and hence likely adapted to use organic substrates at low concentrations [11], it cannot be excluded that sand filter populations develop the capacity to degrade and grow on benzamide at the low concentrations used in this study. This might be suggested by the increased benzamide degradation observed for SFC-SI in water-DI amended with 600 μg/L benzamide. In contrast, in systems that contained 100 μg/L benzamide, degradation of benzamide by the tested SFCs was never observed. Moreover, once established, MSH1 cells might have firmly occupied the niche provided by the selective compound avoiding its use by rare endogenous resident SFC populations especially when lower doses of the compound are used. The use of meta-omics might provide further insight in the adaptation process of the SFC community to benzamide addition.

The modelling results indicated that the BAM degradation was retarded when benzamide was added (Fig. 4D). Since initial BAM and benzamide conversion uses the same BbdA enzyme, benzamide likely competed with BAM for the catalytic site of BbdA [27]. Although the concentration of benzamide in this study was 10 fold higher of that of BAM, the effect looks minimal. The latter can be explained by the Km of BbdA that is 3.5 fold lower for BAM compared to benzamide [27] and hence the BbdA will have a higher affinity for BAM at the micro-concentrations that we use for both compounds.

Earlier sand filter column and pilot system experiments showed that the BAM degradation performance observed after bioaugmentation with MSH1 quickly deteriorated. This was likely due to a limitation of AOC and organic energy carriers in the oligotrophic DWTP systems, leading to loss of cells as well as loss of intrinsic cell activity, possibly exacerbated by the exploitative competition with residents [25]. Our results show that adding an extraneous C-source like benzamide as a selective C-source for MSH1, support bioaugmentation by assisting in establishing a niche for MSH1. However, we expect the selective C-source to come with an additional bonus since it will act as an additional energy-carrier which assists in maintaining activity and hence in long-term functionality of MSH1 in the bioaugmented system. Yet, these scenarios have to be demonstrated in future research and have to be examined in continuous systems as intended to be used for application like in sand filters exploited in DWTPs. We expect that the dosage of the inoculum, the dosage of benzamide or any other selective C-source and the time period of administration of the selective C-source is crucial to avoid outgrowth of endogenous bacteria and to support long term functional maintenance while ensuring the quality and biostability of the finished water [57].

Bioaugmentation resembles an invasion process [2, 5, 46]. Selection has been indicated as a primary determinant of invasion in microbial invasion theories [1, 2] and niche creation is as such expected to be a primary tool in biotechnology for assisting the introduction and establishment of beneficial species in microbial communities [8, 58–60]. Niche creation can be accomplished by several means, such as control of environmental conditions and community evenness [8], reduction of the resident community richness [10], and fluctuations in resource and other disturbances [58–61]. However, these means have been primarily studied in the context of avoiding and understanding invasion but their use in effective applications like in bioaugmentation is less addressed. Moreover, niche creation by adding a selective C-source has not yet been considered as a tool to assist establishment of the added organism and our study provides a first proof of principle. Although it has to be awaited whether a similar process can be designed and be successful for other OMP degraders, our study hence advances on the application of invasion ecology theories and microbial community management for improving the establishment of deliberately introduced beneficial organisms. This work primarily addresses the applications of bioaugmentation in oligotrophic systems, such as treatment of ground waters at DWTPs, but may also be of interest for other biotechnological applications for which introduction and establishment of dedicated beneficial organisms in an already colonized environment is desired. Those include biotechnologies like the use of probiotic inocula in animal and human welfare [62], of plant growth-promoting bacteria in agriculture [63] and of inocula to boost fermentation in food biotechnology [64, 65]. Regardless of which biotechnology is considered, challenges will be to identify suitable carbon sources that are unique for the inocula and that will not lead to the outgrowth of (specific) resident bacteria or alternatively identify suitable dosage strategies. The availability of tools that reliably predict and reconstruct metabolic pathways from genome sequences can assist in finding such selective C-sources [66]. It further has to be awaited whether such a strategy would be efficient in non-oligotrophic biotechnological environments to be managed like soil, the gut, and fermentation broths. Finally, our modelling outcomes indicate that other ecological and biochemical processes might play a role and interfere with the expected effect of the niche creation like interactions with community members or effect of the auxiliary substrate on functionality and hence might have to be taken into account.

## Supplementary Material

Supplementary_material_R3_wraf140

## Data Availability

All data generated or analysed during this study are included in this published article and its supplementary information files. Any source data files are available from the corresponding author upon reasonable request.
