# Supplementary material for: Niche creation improves bioaugmentation of an organic micropollutant degrader in oligotrophic waters
Source: ISME J. 2025 Jul 4;19(1):wraf140. doi: 10.1093/ismejo/wraf140 (PMC12445691; doi:10.1093/ismejo/wraf140)
Supplement: Supplementary_material_R3_wraf140 [file supplementary_material_r3_wraf140.docx]

Supplementary material for

**Niche creation improves bioaugmentation of an organic micropollutant degrader in oligotrophic waters**

Jinsong Wang ^a,†,#^, Bart Raes ^a,†^, Cato Debrabandere ^a^, Veerle van Aken ^a^, Sebastián Jaramillo-Toro ^a^, Steffen Waldherr ^b,^^§^, Benjamin Horemans ^a,b,*^, Dirk Springael ^a,*^

^a^ Division of Soil and Water Management, Department of Earth and Environmental Sciences, KU Leuven, Heverlee B-3001, Belgium

^b^ Chemical Reactor Engineering and Safety (CREaS), Department of Chemical Engineering, KU Leuven, Heverlee B-3001, Belgium

^†^ J. W. and B. R. contributed equally to this paper and are both first authors.

^#^ Present address: Department of Biotechnology, Delft University of Technology, Van der Maasweg 9, 2629 HZ, Delft, the Netherlands

^§^ Present address: Department of Functional and Evolutionary Ecology, University of Vienna, Djerassiplatz 1, 1030 Vienna, Austria

^*^ Corresponding authors:

Dirk Springael ([dirk.springael@kuleuven.be](mailto:dirk.springael@kuleuven.be))

Division of Soil and Water Management, Kasteelpark Arenberg 20 –box 2459, 3001 Leuven, Belgium

tel. +32 16 32 16 04

fax +32 16 32 19 97

Benjamin Horemans ([benjamin.horemans@kuleuven.be](mailto:benjamin.horemans@kuleuven.be))

Division of Soil and Water Management, Kasteelpark Arenberg 20 –box 2459, 3001 Leuven, Belgium

tel. +32 16 32 16 09

fax +32 16 32 19 97

**This supplemental information includes:**

35 pages

5 tables

17 figures

**Document S1**

**Text S1 Detailed information on MSH1 strains.** MSH1-*gfp* is a variant of MSH1 labelled with the *gfp* gene encoding the green fluorescent protein (GFP) and a kanamycin (Km) resistance gene, constructed by introduction of a GFP-2X-miniTn5Km gene cassette [1]. MSH1-*mClover* is a variant of MSH1 labelled with the *mClover3* gene and a gentamycin (Gm) resistance gene, which was delivered by a mini-Tn5 transposon system [2]. To confirm whether the two variants have growth kinetic identical to wild type MSH1, two batch experiments were performed in minimal mineral (MMO) medium containing 1 mM BAM. We first compared the growth of MSH1- *mClover* and wild type MSH1 and then conducted the comparison between MSH1-*gfp* and MSH1-*mClover*. Negative control without inoculum was included. Growth was monitored as OD_600_. Both labelled variants show growth kinetics on BAM that are not statistically different from each other or from the wild type (Figure S1). For all experiments, both variant MSH1 strains were cultured in MMO medium containing 1 mM BAM at 25°C as described previously [3]. When BAM was completely degraded, the culture was centrifuged (6000 × g, 15 min, 18°C) and washed twice with 10 mM assimilable organic carbon (AOC)-restricted MgSO_4_ solution.

**Text S2 Extraction of sand filter community (SFC) cells.** Around 1 kg of sand material was sampled from the top 20 cm of the sand bed and stored in brown glass bottles at 4°C. Prior to the experiment, the indigenous microbial community present in the sand was resuscitated by incubating 15 g of sand in 150 mL MMO medium in an autoclaved Erlenmeyer on an orbital shaker for 3 days at 25 °C (For experiment 4, the incubation time was 1 day). After let settling the sand particles for one hour, the supernatant in the Erlenmeyer was filtered over an autoclaved disposable syringe filter (1.2 μm) (CHROMAFIL Xtra PET-120/25, MACHERY-NAGEL, Germany) to remove larger particles. The filtered supernatant containing the extracted SFC cells was retained for further experiments.

**Text S3 Treatment for removing microbial cells from collected environmental waters and water chemical analysis.** The collected waters were first pasteurized for 1 hour at 60 °C and then filtered through 0.1 µm MCE Membrane filters (MF-Millipore, Sigma-Aldrich, Belgium) to remove indigenous microbes and finally pasteurized a second time for 1h at 60 °C to ensure sterility of the waters. The treated waters were characterized for pH and the concentrations of total organic carbon (TOC), total oxidized nitrogen (TON), orthophosphate and multiple (metal) elements. pH values were determined using a pH meter (Metrohm, Belgium). Total organic carbon (TOC) was measured with a Shimadzu TOC-L CPH (Shimadzu, Japan) equipped with an ASI-L autosampler and a normal sensitivity platinum total carbon catalyst placed in an oven at 680°C. The carrier gas for the analysis with the infrared detector was liquid oxygen (Alphagaz 2 oxygène, Air Liquide, Belgium). Ammonia and nitrate/nitrate concentrations were determined by the modified Berthelot and sulfanilamide colorimetric method, respectively, using a SKALAR-SA40 chemistry automation (Skalar, Netherlands) [4, 5]. Orthophosphate concentrations were spectrophotometrically determined using the molybdate blue method, with absorption at 890 nm measured on a UV-vis spectrophotometer (PerkimElmer Lambda 25 UV-vis spectrometer; Artisan Technology group, USA) [6]. The elemental composition of the waters was determined by Inductively Coupled Plasma Mass Spectroscopy (ICP-MS) (Agilent 7700x, Agilent Technologies, US). Prior to injection, 60 µL of 67% HNO_3_ was added to 6 mL of the 0.1 µm filtered water samples.

**Text S4 Detailed information on growth/biodegradation experiments.**

A first experiment examined the effect of two SFCs on the growth of MSH1 and concomitant BAM degradation in different natural waters. To this end, MSH1-*gfp* was inoculated without and with either SFC-KL or SFC-HA in water-ZW, water-KL, and water-SI spiked with 10 µg/L BAM. In addition, vials containing water-ZW, water-KL, and water-SI spiked with BAM and only inoculated with either SFC-KL or SFC-HA were included.

A second experiment investigated whether benzamide is a selective carbon source for MSH1. The growth kinetics of MSH1-*gfp* and SFC-SI on benzamide were respectively determined in water-DI spiked with benzamide at concentrations of 0, 100, and 600 µg/L. The vials were inoculated with either MSH1 or SFC-SI.

A third experiment examined the effects of benzamide on MSH1 growth and concomitant BAM degradation and on the growth of SFCs. MSH1-*gfp* and SFC-SI were separately inoculated in water-DI spiked with both BAM (10 µg/L) and benzamide (130 µg/L). For comparison, glucose, as a non-aromatic competitive C-source usable by both MSH1 and the SFC, was added to water-DI instead of benzamide, at a C-concentration (90 µg-C/L) equal to this of benzamide.

A final experiment explored how the addition of benzamide affected MSH1 growth and BAM degradation in the presence of SFC under different competitive conditions. To this end, MSH1-*mClover* and SFC-SI was co-inoculated in water-DI without additional carbon source and in water-DI amended with 90 µg-C/L benzamide. We hypothesized that the addition of benzamide will provide a more significant benefit when a more intense competition for a common carbon source exists between MSH1 and SFCs. The diverse competition strengths were established by providing four different concentrations of glucose (i.e., 0, 30, 90, and 200 µg-C/L glucose) that can be used by both MSH1 and the SFC. In addition, four control groups only containing MSH1 were included, in which benzamide and glucose were added with either 0 µg-C/L or 90 µg-C/L (Table S1). As such, in total, 12 test groups were included (Table S1). BAM was always added at a concentration of 10 µg/L (4.4 µg-C/L) in each group.

**Text S5 Detailed description of plate count method and flow cytometry (FC) analysis.** MSH1-*gfp* cell densities were determined by Colony Forming Unit (CFU) counting by plating undiluted, 1:10, 1:100, and 1:1000 diluted samples taken from the experimental vials on R2A agar plates containing 85.8 µM Km. Each replicate was plated three times and the plates were incubated in a dark room at 25°C. After eight days, CFUs were counted that showed green fluorescence on a Blue Light Transilluminator (Safe Imager 2.0, Invitrogen, USA).

Cell densities in vials containing only MSH1-*gfp* and total cell densities (SFCs plus MSH1-*gfp*) were determined with a BD Accuri C6 FC equipped with a C6 autosampler and 50 mW solid state laser (BD Accuri cytometers, Belgium) as previously described [3]. In experiment 4, the total cell densities as well as the MSH1-*mClover* densities were quantified by a CytoFLEX S FC coupled with 405, 488, and 561 nm lasers (Beckam Coulter, USA). For determining the total cell density (SFCs plus MSH1-*mClover*), samples (200 µL for each) were stained with 2 µL SYBR green propidium iodide (SGPI) and incubated for 20 min in the dark at 37 °C. For quantifying MSH1-*mClover* cell density, samples (200 µL for each) were used without staining*.* In both cases, the fluorescence intensities were determined by excitation using the 488 nm laser and collecting emission with the 525 nm filter. For cell densities higher than 2×10^5^ cells/mL, samples were diluted appropriately with 0.1 µm filtered Evian bottled mineral water prior to FC analysis. The preparation of SGPI was described previously [3].

**Text S6 RP-UHPLC-MS/MS analysis.** BAM concentrations were determined by injecting 10 µL samples on a Nexera apparatus (Shimadzu Corp, Japan) equipped with a LC-MS8045 triple quadrupole mass spectrometer and a Triart C18 column (150 × 3 mm, 3 µm; YMC) kept at 40°C as previously reported [3].

Benzamide concentrations were determined on the same apparatus as used for determining BAM concentrations and equipped with the same column but applying an injection volume of 50 µL and elution using an isocratic flow (0.4 mL/min) of 70% methanol and 30% ultrapure water acidified with 1% formic acid and 2 mM of ammonium formate (pH = 2.8). For mass spectrometry, the interface, desolvation line and heat block temperatures were kept at 300°C, 300°C and 500°C, respectively. Nebulizing, drying and heating gas flow were set at 3.0, 15.0 and 5.0 L/min, respectively. Full MS and MS^2^ scans were conducted in the positive ionization mode and the transition ions of *m/z* 77.05, 79.10 and 105.20 were monitored with applied collision energies of -26, -14 and -18 V, respectively. For BAM, the LOD and LOQ were 0.05 µg/L and 0.15 µg/L, respectively. For benzamide, the LOQ were 10 µg/L and the LOD was not determined. The calibration curve had a good linearity (*R*^2^>0.99) (results not shown).

**Text S7 Calculation and Interpretation of model evaluation statistics (Error Index).**

**Relative Mean Absolute Error (RMAE)**

The relative mean absolute error (RMAE) quantifies the average magnitude of model prediction errors relative to the mean of the observed values. It is unitless and enables direct comparison of model performance across datasets with different scales. RMAE was calculated as:

$$RMAE (\%)=\frac{1}{n}\sum_{i=1}^{n} \frac{\left| m_{exp, i}-m_{pred,i} \right|}{\bar{m_{exp}}}\times100$$

where $m_{exp, i}$ and $m_{pred,i}$ are the experimental and predicated values at index $i$, and $\bar{m_{exp}}$ is the mean of experimental values. Interpretation benchmarks are described as below:

RMAE < 10%: Excellent fit”;

RMAE = 10–20%: Good fit;

RMAE = 20–30%: Moderate fit;

RMAE >30%: Poor fit.

**Percent Bias (PBIAS)**

Percent bias (PBIAS) evaluates the average tendency of the model to overestimate or underestimate experimental values, expressed as a percentage. PBIAS was calculated as:

$$PBIAS=\frac{\sum_{i=1}^{n} (m_{exp,i}-m_{pred,i})}{\sum_{i=1}^{n} m_{exp,i}}\times100$$

where $m_{exp, i}$ and $m_{pred,i}$ are the experimental and predicated values at index $i$. Negative PBIAS values indicate model overestimation; positive values indicate underestimation. It should be noted that the overestimation of residual BAM/benzamide concentration means the underestimation of BAM/benzamide degradation and vice versa.

**Cosine Distance**

Cosine distance assesses the similarity in shape (trend) between experimental and predicted data vectors, independent of magnitude. Cosine distance was calculated as:

$$Consine distance=1-\frac{\sum_{i=1}^{n} m_{exp,i}m_{pred,i}}{\sqrt{\sum_{i=1}^{n} m_{exp,i}^{2}}\cdot\sqrt{\sum_{i=1}^{n} m_{pred,i}^{2}}}$$

where $m_{exp, i}$ and $m_{pred,i}$ are the experimental and predicated values at index $i$. Lower values indicate greater similarity in trend between predicted and observed values. Interpretation benchmarks are proposed as below:

Cosine distance < 0.01: Excellent;

Cosine distance = 0.01–0.05: Very good;

Cosine distance = 0.05–0.15: Moderate;

Cosine distance > 0.15: Poor.

**Document S2**

**Supplementary tables**

**Table S1** Overview of the design of the final experiment that explored the effect of auxiliary C-source on growth of MSH1 and concomitant BAM degradation in the presence of SFC-SI.

| **Group number** | **Microbial community** | **Concentrations of added carbon sources (µg-C/L)**^a^ | | | **Figure**  **number** |
| --- | --- | --- | --- | --- | --- |
|  |  | Benzamide | Glucose | BAM |  |
| 1 | MSH1+SFC | 0 | 0 | 4.4 | 4a |
| 2 | MSH1+SFC | 0 | 30 | 4.4 | - |
| 3 | MSH1+SFC | 0 | 90 | 4.4 | 4e |
| 4 | MSH1+SFC | 0 | 200 | 4.4 | - |
| 5 | MSH1+SFC | 90 | 0 | 4.4 | 4b |
| 6 | MSH1+SFC | 90 | 30 | 4.4 | - |
| 7 | MSH1+SFC | 90 | 90 | 4.4 | 4f |
| 8 | MSH1+SFC | 90 | 200 | 4.4 | - |
| 9 | MSH1 (*Control*) | 0 | 0 | 4.4 | 4c |
| 10 | MSH1 (*Control*) | 0 | 90 | 4.4 | 4g |
| 11 | MSH1 (*Control*) | 90 | 0 | 4.4 | 4d |
| 12 | MSH1 (*Control*) | 90 | 90 | 4.4 | 4h |

(a: the concentrations were normalized as µg-C/L. For benzamide, 90 µg-C/L was equal to 130 ug/L; for BAM, 4.4 µg-C/L was equal to 10 ug/L; for glucose, 30, 90, and 200 µg-C/L were equal to 75, 225, and 500 ug/L, respectively.)

**Table S2.** Definition of the kinetic parameters used in the equations shown in Table 1.

| **Parameter** | **Definition** | **Parameter value** | **Parameter unit** | **Parameter value source**^g^ |
| --- | --- | --- | --- | --- |
| $X_{m}$ | MSH1 biomass density | Experiment dependent | cells/L | Experimental data from exp. 4 |
| $X_{m, Final}$ | MSH1 biomass density at the end of experiment | Experiment dependent | cells/L | Experimental data from exp. 4 |
| $X_{m, Max}$ | The maximum MSH1 biomass density during experiments | Experiment dependent | cells/L | Experimental data from exp. 4 |
| $X_{s}$ | SFC biomass density | Experiment dependent | cells/L | Experimental data from exp. 4 |
| $S_{\mathrm{BAM}}$ | BAM substrate concentration | Experiment dependent | µg-C/L | Experimental data |
| $Q_{\mathrm{BAM}}$ | Degradation rate of BAM | 1.36×10^-9^ | hour^-1^‧(cells/L)^-1^ | Estimated from experiment 4^c^ |
| $S_{\mathrm{mAOC}}$ | AOC substrate concentration available to MSH1 (AOC_MSH1_) | Experiment dependent | µg-C/L | Estimated from experiment 4^a^ |
| $Y_{\mathrm{mAOC}}$ | Yield of MSH1 on AOC_MSH1_ | 1.00×10^7^ | cells/µg-C | Fixed AOC conversion factor^b^ |
| $R_{\mathrm{mAOC}}$ | Maximum growth rate of MSH1 on AOC_MSH1_ | 1.37×10^-2^ | hour^-1^ | Estimated from experiment 4^c^ |
| $K_{\mathrm{mAOC}}$ | Half-saturation coefficient of MSH1 on AOC_MSH1_ | 2.59×10^-2^ | µg-C/L | Estimated from experiment 4^c^ |
| $S_{\mathrm{sAOC}}$ | AOC substrate concentration available to SFC (AOC_SFC_) | Experiment dependent | µg-C/L | Estimated from experiment 4^a^ |
| $Y_{\mathrm{sAOC}}$ | Yield of SFC on AOC_SFC_ | 1.00×10^7^ | cells/µg-C | Fixed AOC conversion factor^b^ |
| $R_{\mathrm{sAOC}}$ | Maximum growth rate of SFC on AOC_SFC_ | 0.10 | hour^-1^ | Estimated from experiment 4^c^ |
| $K_{\mathrm{sAOC}}$ | Half-saturation coefficient of SFC on AOC_SFC_ | 3.45×10^2^ | µg-C/L | Estimated from experiment 4^c^ |
| $S_{\mathrm{Benz}}$ | Benzamide substrate concentration | Experiment dependent | µg-C/L | Experimental data |
| $Y_{\mathrm{Benz}}$ | Yield of MSH1 on benzamide | 1.05×10^6^ | cells/µg-C | Estimated from experiment 4^c^ |
| $R_{\mathrm{Benz}}$ | Maximum growth rate of MSH1 on benzamide | 7.88×10^-2^ | hour^-1^ | Estimated from experiment 4^c^ |
| $K_{\mathrm{Benz}}$ | Half-saturation coefficient of MSH1 on benzamide | 1.83×10^-4^ | µg-C/L | Estimated from experiment 4^c^ |
| $S_{\mathrm{Glc}}$ | Glucose substrate concentration | Experiment dependent | µg-C/L | Estimated from experiment 4^d^ |
| $Y_{\mathrm{mGlc}}$ | Yield of MSH1 on glucose | 6.71×10^5^ | cells/µg-C | Estimated from experiment 4^c^ |
| $R_{\mathrm{mGlc}}$ | Maximum growth rate of MSH1 on glucose | 7.46×10^-2^ | hour^-1^ | Estimated from experiment 4^c^ |
| $K_{\mathrm{mGlc}}$ | Half-saturation coefficient of MSH1 on glucose | 1.90 | µg-C/L | Estimated from experiment 4^c^ |
| $Y_{\mathrm{sGlc}}$ | Yield of SFC on glucose | 1.88×10^6^ | cells/µg-C | Estimated from experiment 4^c^ |
| $R_{\mathrm{sGlc}}$ | Maximum growth rate of SFC on glucose | 5.06×10^-2^ | hour^-1^ | Estimated from experiment 4^c^ |
| $K_{\mathrm{sGlc}}$ | Half-saturation coefficient of SFC on glucose | 14.72 | µg-C/L | Estimated from experiment 4^c^ |
| $D_{m}$ | MSH1 decay rate | Experiment dependent | hour^-1^ | Estimated from experiment 4^e^ |
| $D_{s}$ | SFC decay rate | 0.00 | hour^-1^ | Estimated from experiment 4^f^ |

(**a:** the concentrations of AOC were calculated based on the net biomass growth on AOC with a fixed AOC conversion factor of 1×10^7^; **b:** the fixed value was reported previously [7]; **c:** the values of these parameters were obtained from a pre-estimation with the experimental data from a specific group of experiment 4 using the biokinetic model framework adapted from a previous study [8] The detailed procedure of pre-estimation was given in Figure S3; **d:** the concentrations of glucose were calculated based on the net biomass growth on glucose, in which the growth yields on glucose were used as the conversion factor; **e:** the decay rate of MSH1 was estimated from the experimental data using the a first-order decay model (Eq. (8)) in Table 1; **f:** this value was set as 0, as no decay was observed for the SFC; **g:** in this column, experiment 4 (exp. 4) refers to the final experiment that explored the effect of auxiliary C-source on growth of MSH1 and concomitant BAM degradation in the presence of an SFC.)

**Table S3.** Values of determined chemical parameters in the four used freshwater samples.

| **Water sample** | **Orthophosphate**  **(µg/L)** | **TON^a^**  **(µg/L)** | **TOC^b^**  **(µg/L)** | **pH** |
| --- | --- | --- | --- | --- |
| Water-DI | 251.17 | 11179 | 1942 | 8.4 |
| water-ZW | 61.17 | 1700 | 2578 | 8.5 |
| Water-KL | 1.17 | 1037 | 3048 | 7.5 |
| Water-SI | 2.83 | 1255 | 2651 | 7.5 |

(a: refer to Total Oxidised Nitrogen (TON), which is the sum of nitrate and nitrite; b: refer to Total Organic Carbon (TOC).)

**Table S4.** Elemental concentration in the used freshwater samples.

| **Water sample** | **Elemental concentration (mg/L)** | | | | **Elemental concentration (µg/L)** | | | | | | | | | | | | | |
| --- | --- | --- | --- | --- | --- | --- | --- | --- | --- | --- | --- | --- | --- | --- | --- | --- | --- | --- |
|  | **Na** | **Mg** | **K** | **Ca** | **Al** | **P** | **V** | **Cr** | **Mn** | **Fe** | **Co** | **Ni** | **Cu** | **Zn** | **As** | **Mo** | **Sb** | **Pb** |
| Water-DI | 49.63 | 11.06 | 4.83 | 100.01 | 1.65 | 251.03 | 2.06 | 2.18 | 4.89 | 2.91 | 0.19 | 1.34 | 1.04 | 7.85 | 1.07 | 2.22 | 0.19 | 0.35 |
| water-ZW | 33.14 | 4.60 | 4.56 | 72.74 | 3.15 | 93.53 | 1.78 | 17.08 | 1.03 | 3.61 | 0.17 | 1.11 | 1.13 | 5.94 | 1.07 | 0.45 | 0.37 | 0.12 |
| Water-KL | 40.49 | 7.62 | 7.66 | 80.39 | 5.89 | 9.41 | 0.68 | 1.08 | 71.04 | 6.13 | 0.10 | 1.58 | 2.83 | 32.10 | 0.76 | 1.58 | 0.16 | 0.12 |
| Water-SI | 26.20 | 3.24 | 1.43 | 91.43 | 3.75 | 11.01 | 0.54 | 0.47 | 157.25 | 11.26 | 0.03 | 0.62 | 0.28 | 13.74 | 0.58 | 0.28 | 0.02 | 0.09 |

**Table S5.** Summary of relative mean absolute error (RMAE), percent bias (PBIAS), and cosine distance for model predictions compared to observed experimental data.

| Operation Scenario | Simulated Parameter | RMAE^a^ (%) | PBIAS (%) | Cosine Distance | Figure  Number^b^ |
| --- | --- | --- | --- | --- | --- |
| MSH1 + SFC  *(only BAM)* | MSH1 cell density | 36.3 | 24.3 | 0.11 | 4a |
|  | SFC cell density | 26.6 | 2.8 | 0.03 | S17a |
|  | BAM concentration | 32.4 | 32.6 | 0.07 | 4a |
|  | Benzamide concentration | N.A.^c^ | N.A. | N.A. | S16a |
| MSH1 + SFC  *(BAM*  *+ Benzamide)* | MSH1 cell density | 25.0 | -4.4 | 0.03 | 4b |
|  | SFC cell density | 27.2 | 13.3 | 0.04 | S17b |
|  | BAM concentration | 44.1 | 43.9 | 0.14 | 4b |
|  | Benzamide concentration | 9.4 | -5.1 | 0.00 | S16b |
| Only MSH1  *(only BAM)* | MSH1 cell density | 12.8 | 8.1 | 0.01 | 4c |
|  | SFC cell density | N.A. | N.A. | N.A. | S17c |
|  | BAM concentration | 15.2 | 5.4 | 0.01 | 4c |
|  | Benzamide concentration | N.A. | N.A. | N.A. | S16c |
| Only MSH1  *(BAM*  *+ Benzamide)* | MSH1 cell density | 27.2 | -4.1 | 0.04 | 4d |
|  | SFC cell density | N.A. | N.A. | N.A. | S17d |
|  | BAM concentration | 46.9 | 46.5 | 0.14 | 4d |
|  | Benzamide concentration | 20.3 | -6.6 | 0.02 | S16d |
| MSH1 + SFC  *(BAM*  *+ Glucose)* | MSH1 cell density | 135.3 | -135.3 | 0.03 | 4e |
|  | SFC cell density | 56.7 | -24.9 | 0.06 | S17e |
|  | BAM concentration | 59.5 | 59.3 | 0.23 | 4e |
|  | Benzamide concentration | N.A. | N.A. | N.A. | S16e |
| MSH1 + SFC  *(BAM*  *+ Benzamide*  *+ Glucose)* | MSH1 cell density | 58.2 | 14.4 | 0.17 | 4f |
|  | SFC cell density | 52.5 | -17.3 | 0.07 | S17f |
|  | BAM concentration | 51.0 | 51.3 | 0.20 | 4f |
|  | Benzamide concentration | 10.5 | 10.5 | 0.01 | S16f |
| Only MSH1  *(BAM*  *+ Glucose)* | MSH1 cell density | 15.8 | -5.4 | 0.02 | 4g |
|  | SFC cell density | N.A. | N.A. | N.A. | S17g |
|  | BAM concentration | 40.0 | 40.4 | 0.09 | 4g |
|  | Benzamide concentration | N.A. | N.A. | N.A. | S16g |
| Only MSH1  *(BAM*  *+ Benzamide*  *+ Glucose)* | MSH1 cell density | 37.3 | 25.8 | 0.04 | 4h |
|  | SFC cell density | N.A. | N.A. | N.A. | S17h |
|  | BAM concentration | 53.9 | 53.5 | 0.21 | 4h |
|  | Benzamide concentration | 2.7 | 2.7 | 0.00 | S16h |

(**a**: The relative mean absolute error (RMAE) was calculated as the mean absolute error (MAE) divided by the mean of the observed data, and is reported as a percentage to allow assessment of model accuracy independent of the absolute value scale; **b**: the figure includes the experimental data and simulated curves for each parameter; **c**: “N.A.” refers to “Not Applicable”.)

**Document S3**

**Supplementary figures.**

**
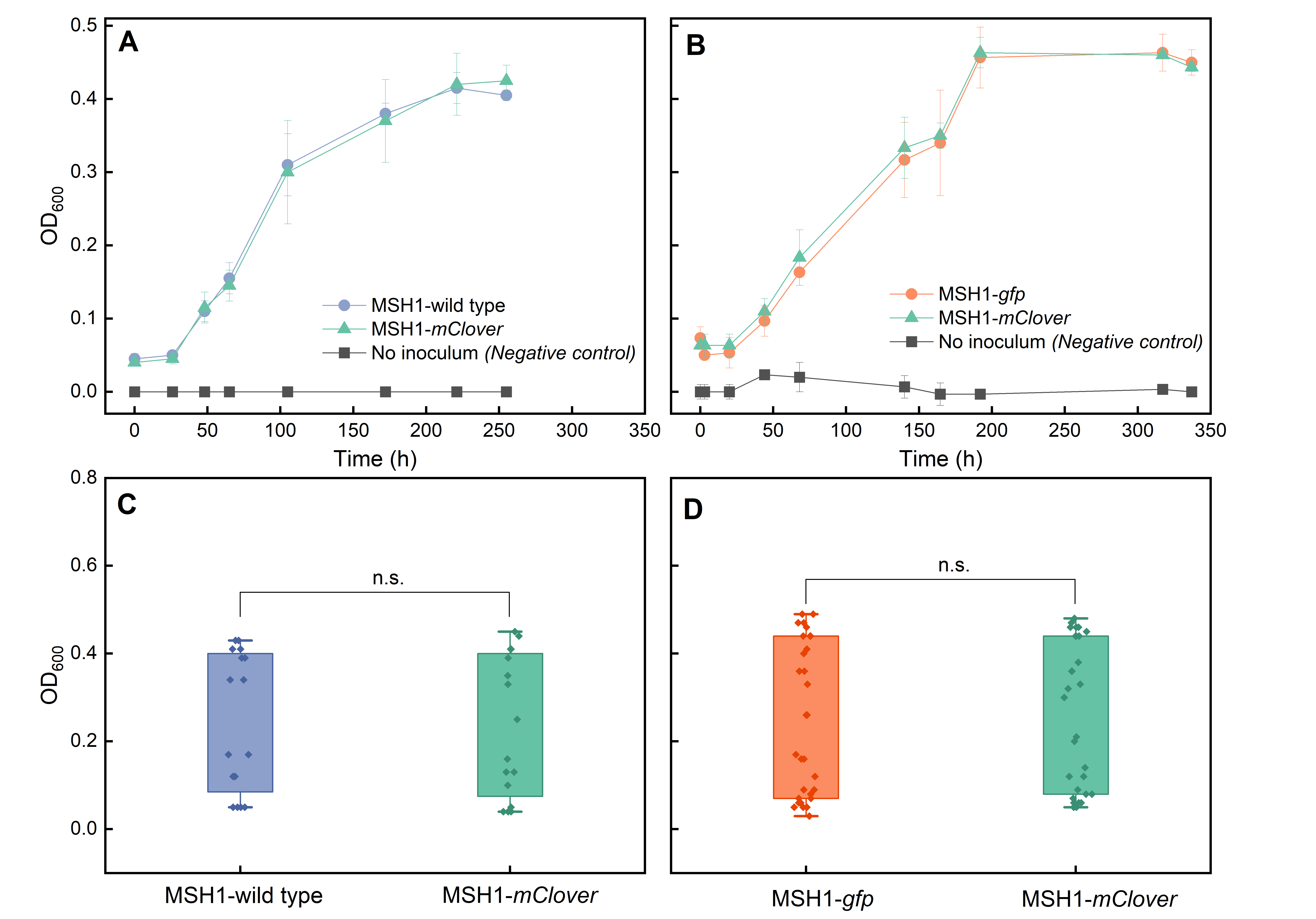
**

**Figure S1.** Comparison of the growth kinetics for MSH1-*gfp*, MSH1- *mClover*, and MSH1-wild type. (**A**) growth curves for MSH1-wild type and MSH1- *mClover*, (**B**) growth curves for MSH1-*gfp* and MSH1- *mClover*, (**C**) statistical difference in the OD_600_-based cell density between MSH1-wild type and MSH1- mClover, (**D**) statistical difference in the OD_600_-based cell density between MSH1-*gfp* and MSH1- mClover. A *t*-test was used to analyse the significance of differences. “n.s.” indicates no significant difference with *P* value>0.05.

**
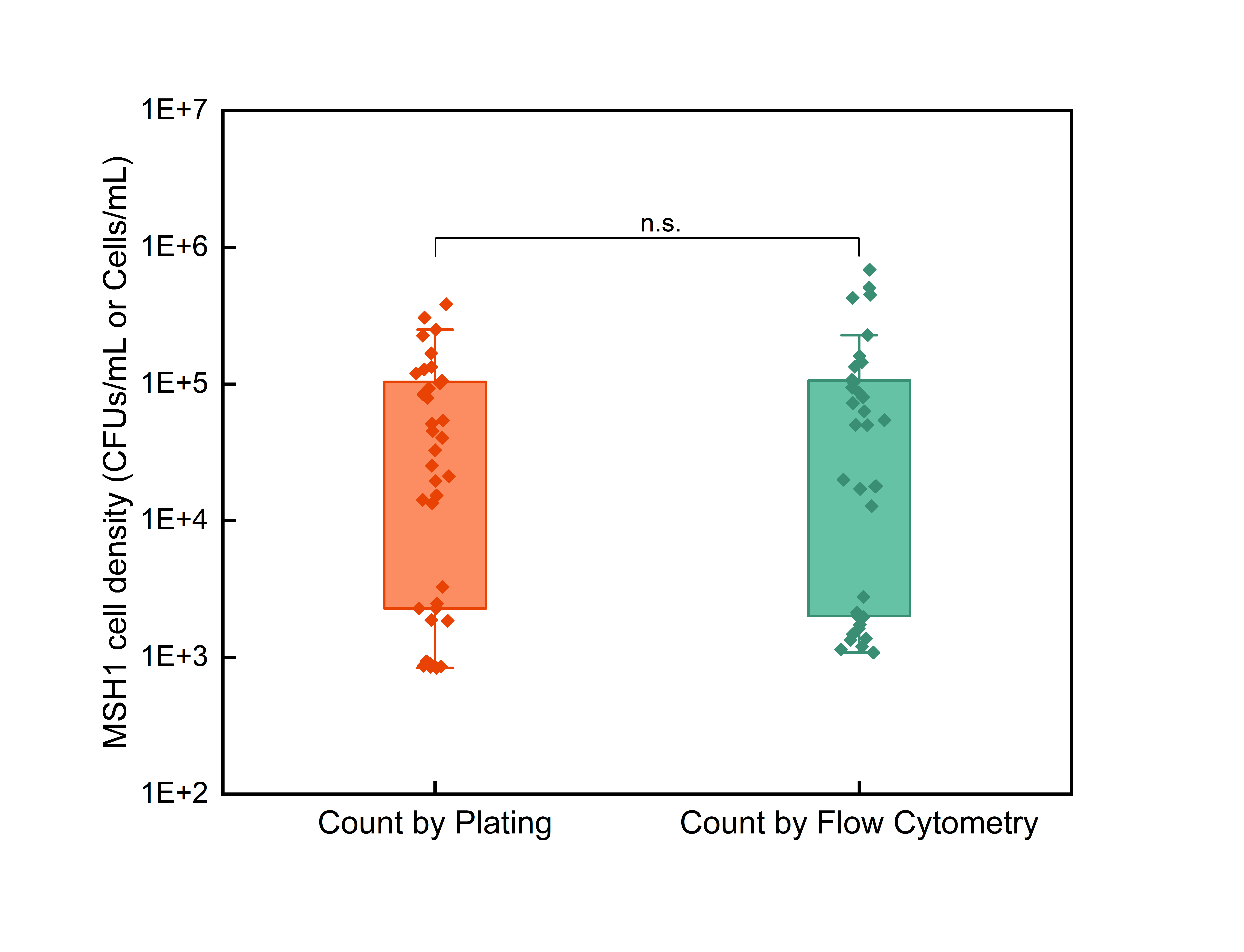
**

**Figure S2.** Comparison of MSH1-*gfp* cell density determined by plating (CFUs/mL) and flow cytometry (cells/mL). Cell counts were measured under conditions containing only MSH1-*gfp*. No significant difference was observed between the two quantification methods (*t*-test, *P* value=0.30), indicating consistency between culture-based and cytometric enumeration. Data are presented on a log scale, with individual replicates (n=36) shown and box plots indicating the interquartile range.

**
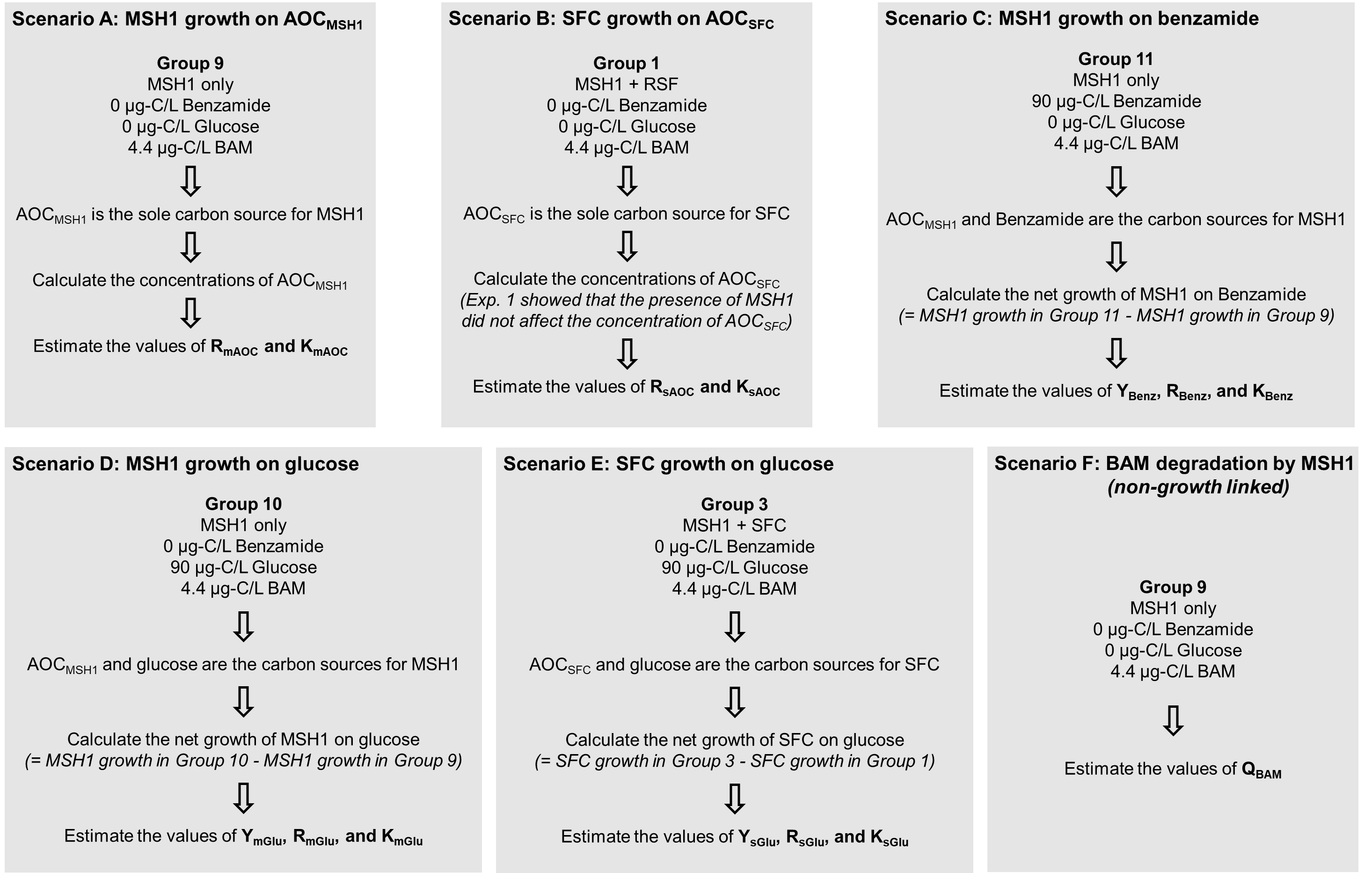
**

**Figure S3** Used scenarios for predicting the kinetic parameters used in the model simulation for predicting the kinetics of biomass growth (SFC and MSH1) and BAM/benzamide degradation in higher complexity conditions. The simulated trajectories are shown in Figure S15.

**
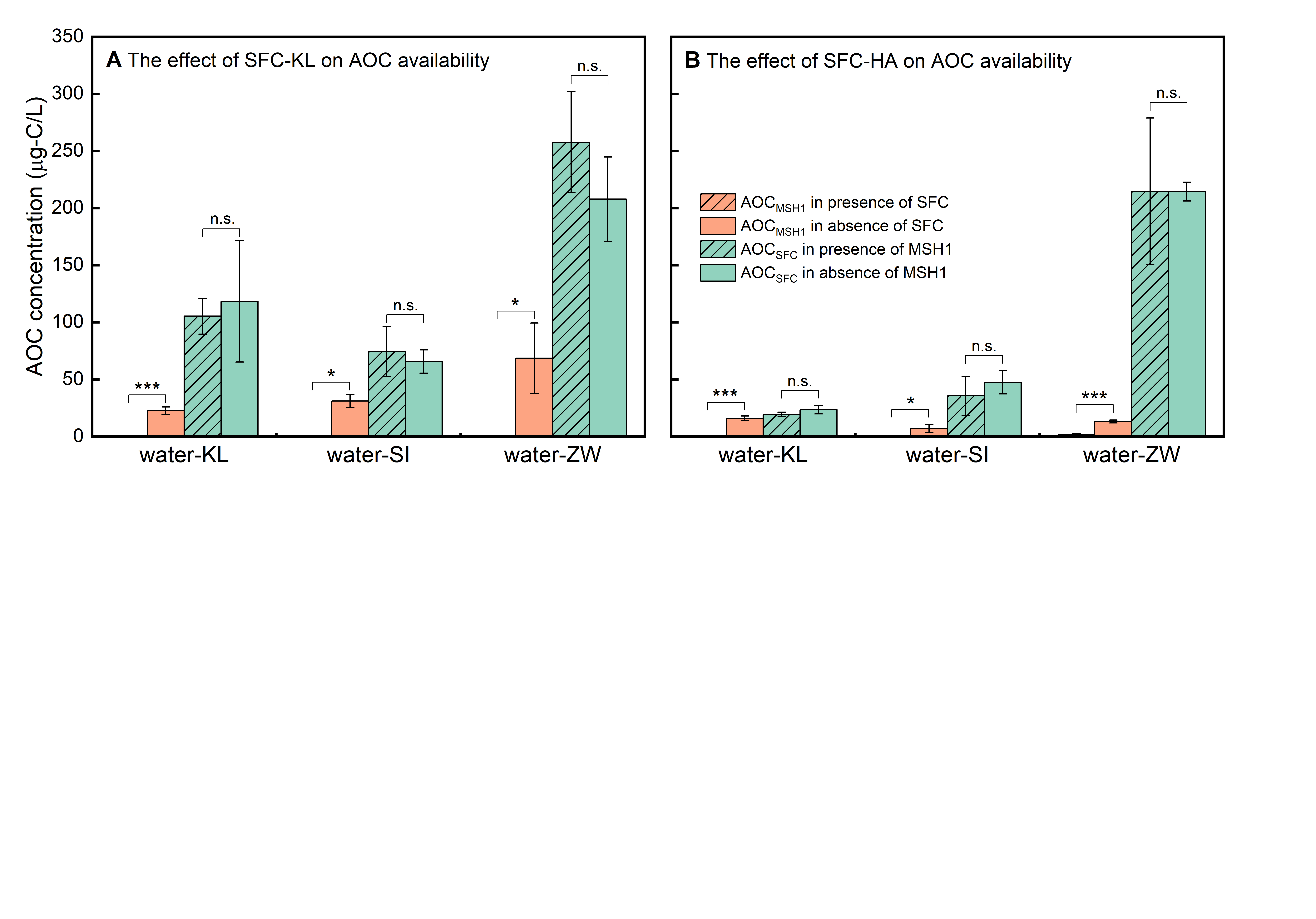
**

**Figure S4** The effects of the presence of (**A**) SFC-KL and (**B**) SFC-HA on the AOC available for MSH1 (AOC_MSH1_) and SFC (AOC_SFC_) in three freshwaters, i.e., water-KL, water-SI, and water-ZW. A *t*-test was used to analyse the significance of differences in AOC concentrations between groups, “n.s.” indicates no significant difference with *P* value>0.05; “*” means *P* value is 0.01–0.05; “***” means *P* value is 0.0001–0.001. Error bars represent standard deviation of three biological replicates.


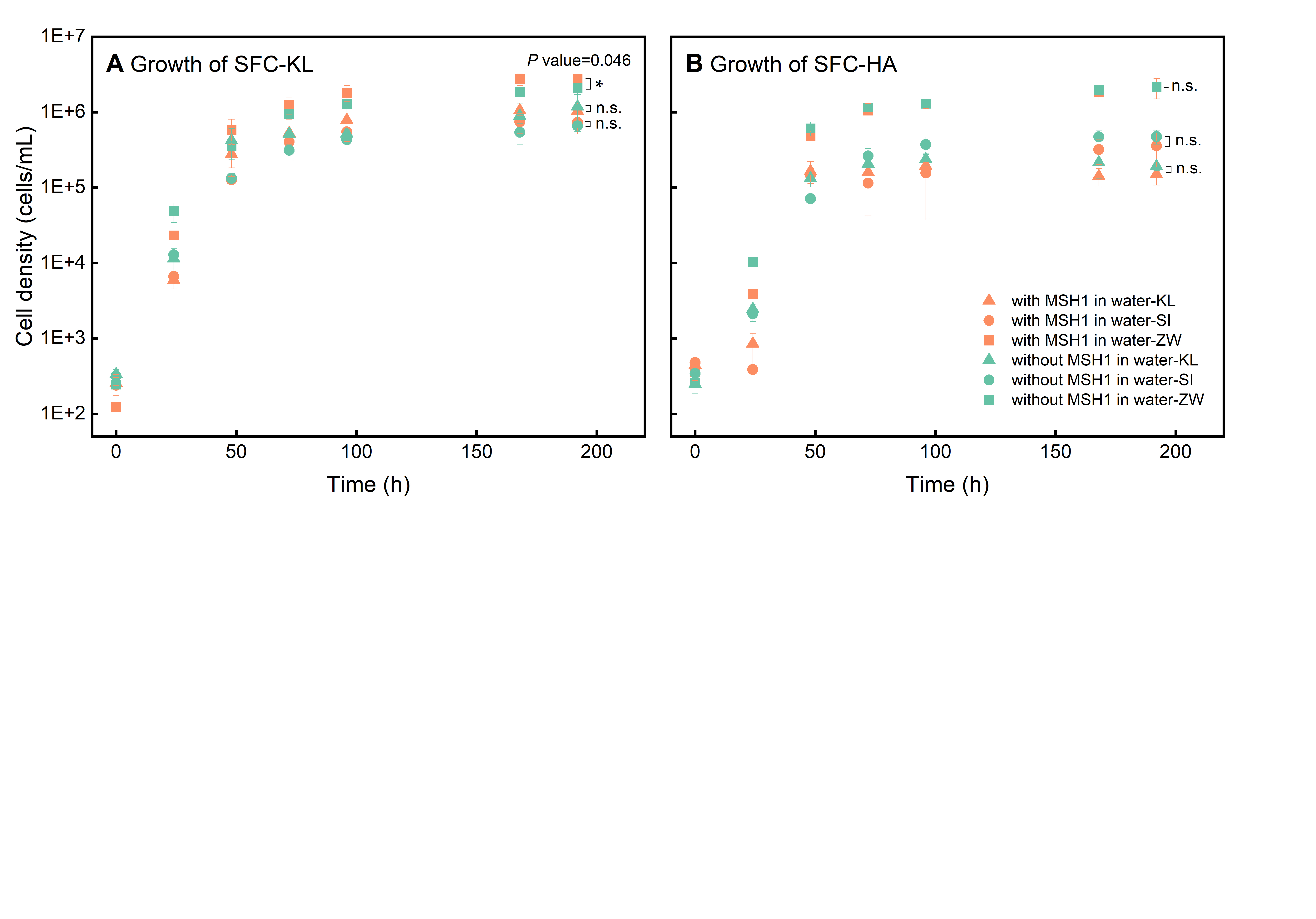


**Figure S5** Growth of SFC-KL and SFC-HA in the experiment that assesses the effect of the presence of an SFC on growth of MSH1 and concomitant BAM degradation in three freshwaters including water-KL, water-SI, and water-ZW. (**A**) growth of SFC-KL and (**B**) growth of SFC-HA. A *t*-test was used to analyse the significance of differences in SFC cell density between groups, “n.s.” indicates no significant difference with *P* value>0.05; “*” means *P* value is 0.01–0.05. Error bars represent standard deviation of three biological replicates.


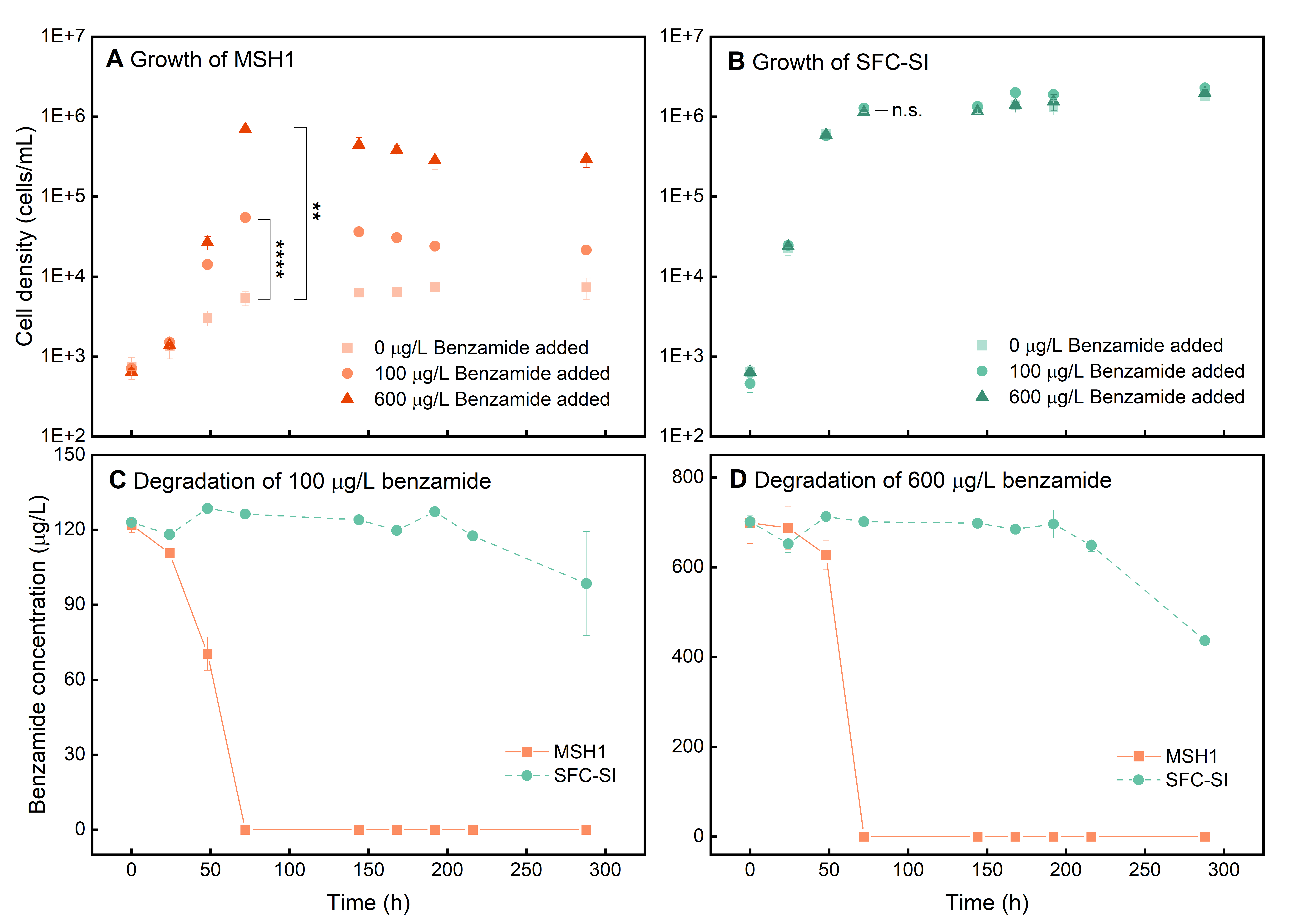


**Figure S6** Growth of MSH1 and SFC-SI on benzamide supplemented at concentrations of 0, 100, and 600 µg/L in DI-water in the experiment that assesses the utilisation of benzamide as a selective C-source for MSH1. (**A**) growth of MSH1; (**B**) growth of SFC-SI; (**C**) degradation of benzamide supplemented at a concentration of 100 µg/L in the presence of MSH1 or SFC-SI; (**D**) degradation of benzamide added at a concentration of 600 µg/L in the presence of MSH1 or SFC-SI. A *t*-test was used to analyse the significance of differences in MSH1/SFC cell density between groups, “n.s.” indicates no significant difference with *P* value>0.05; “**” means *P* value is 0.001–0.01; “****” means *P* value is < 0.0001. Error bars represent standard deviation of three biological replicates.

**
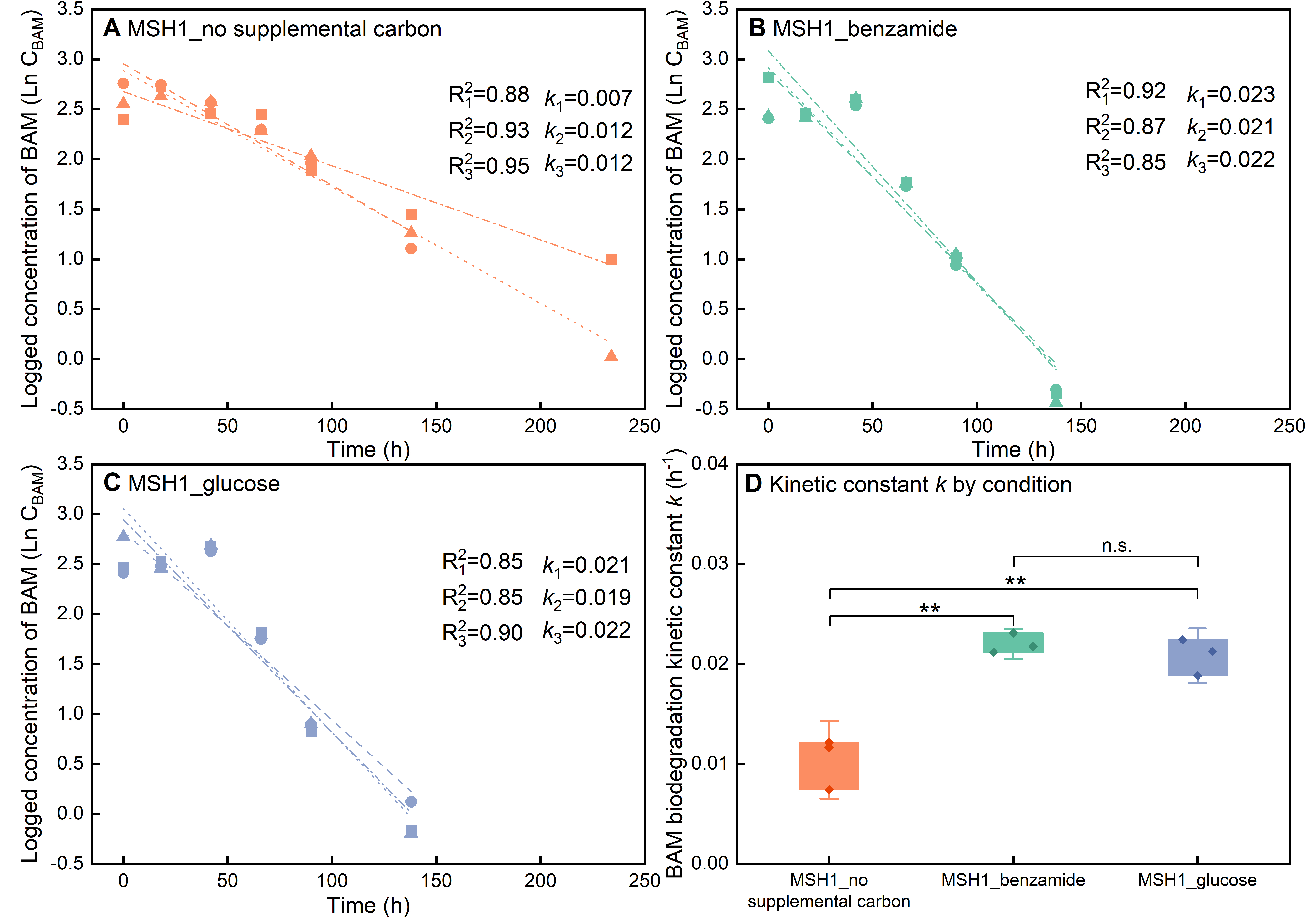
**

**Figure S7.** Kinetics of BAM biodegradation by MSH1 in the experiment that explored the effect of an auxiliary C-source (either benzamide or glucose) on BAM degradation in absence of SFCs: (**A**) without supplemental carbon source, (**B**) with benzamide, and (**C**) with glucose. The circle, triangle and square symbols represent the triplicate measured data while the three dot curves show the fitting results obtained from linearized first-order kinetic modelling. The coefficients of determination (*R*^2^) and kinetic constants (*k*) are shown individually for each triplicate in the figure. (**D**) BAM biodegradation kinetic constants under different conditions. A *t*-test was used to analyse the significance of differences in BAM biodegradation first order kinetic constants between groups as calculated from linearized first-order kinetic modelling in panels a–c, “n.s.” indicates no significant difference with *P* value>0.05; “**” means *P* value is 0.001–0.01.


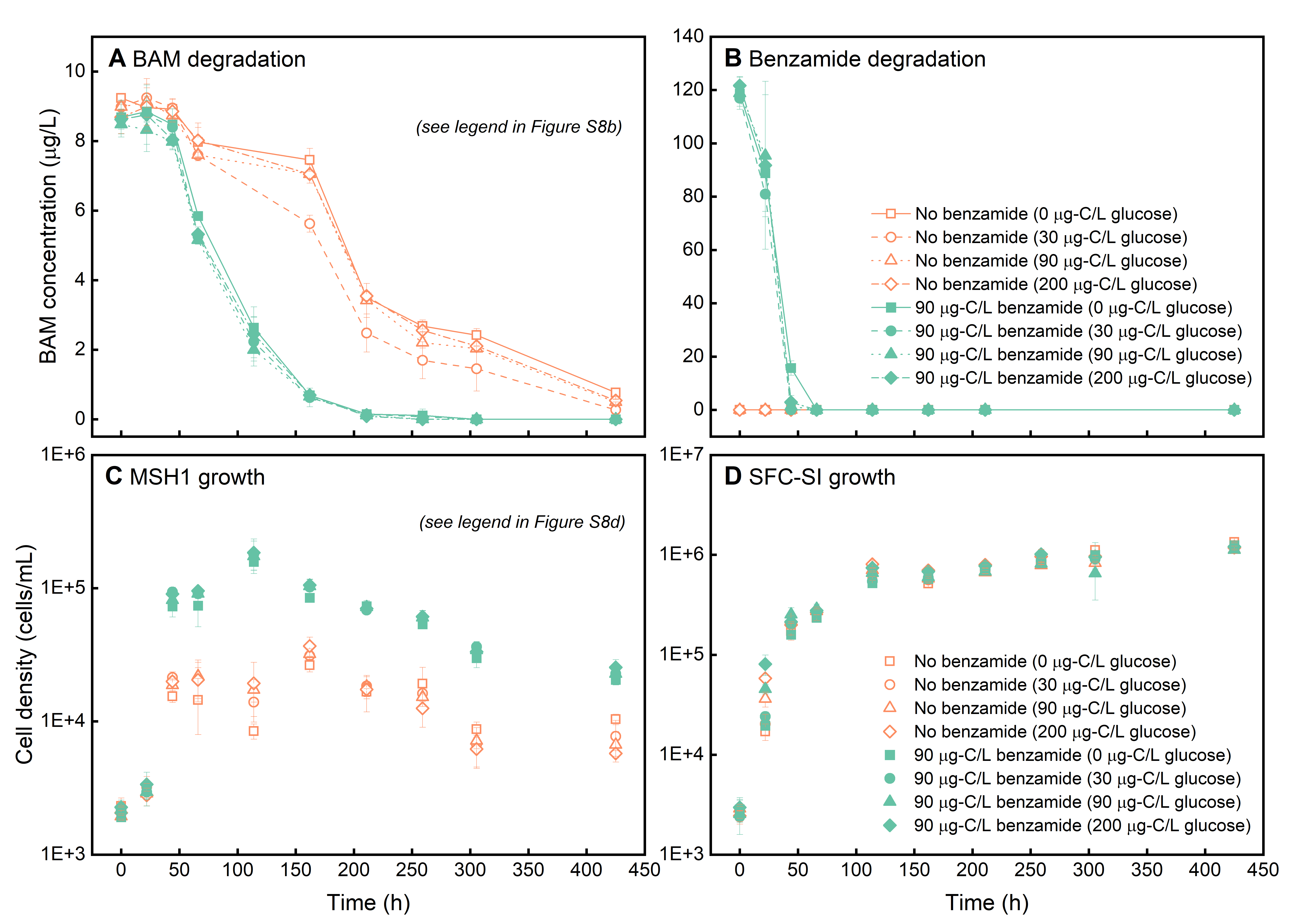


**Figure S8.** Effect of benzamide and glucose on growth of MSH1 and concomitant BAM biodegradation in the presence of SFC-SI. Biodegradation of (**A**) BAM and (**B**) benzamide and growth of (**C**) MSH1 and (**D**) SFC-SI in DI-water amended with and without benzamide at different concentrations of glucose. Error bars represent standard deviation of three biological replicates.

**
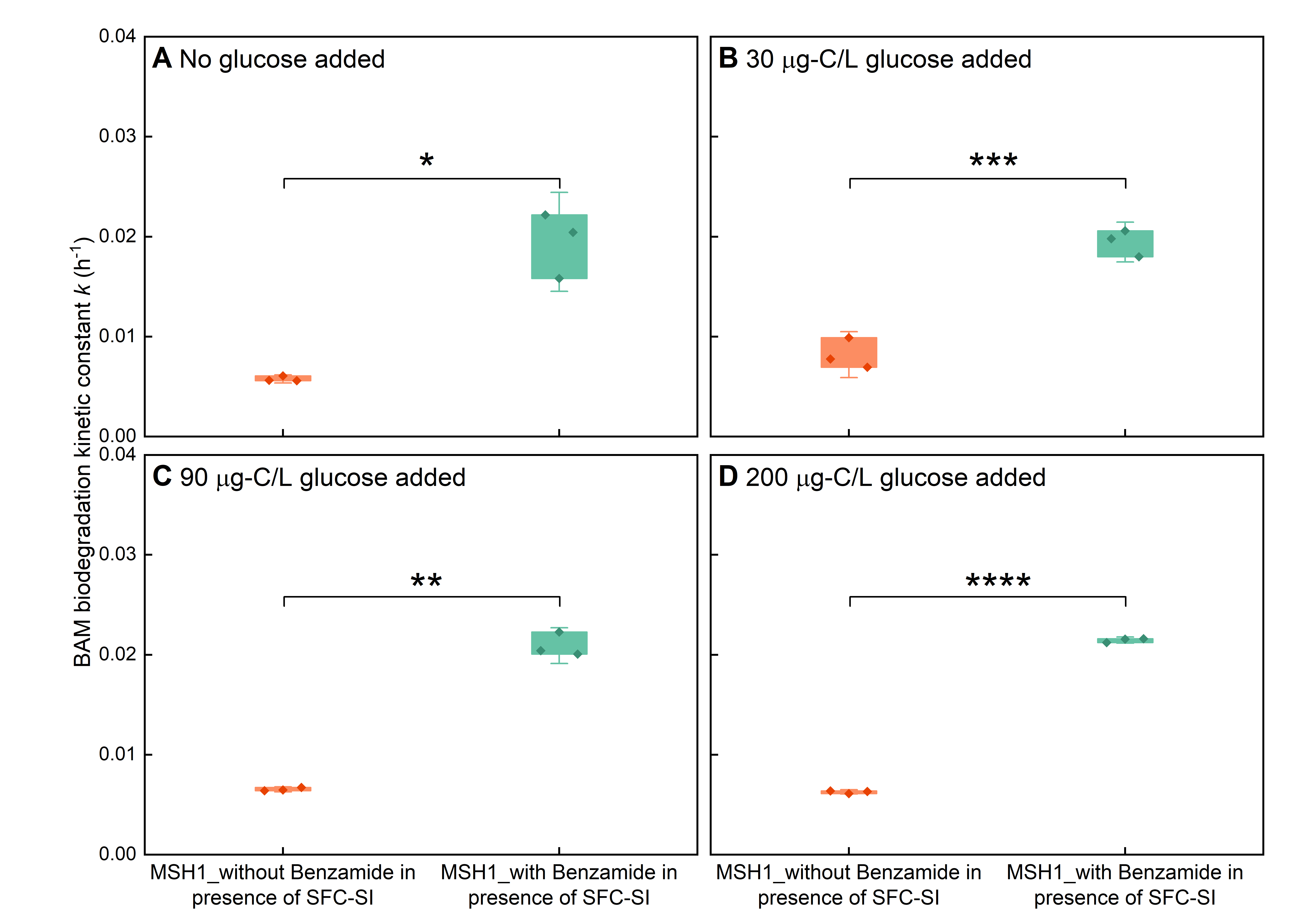
**

**Figure S9.** Statistically significant difference in BAM biodegradation kinetic constants between the condition with benzamide and the one without benzamide in presence of SFC-SI under different concentrations of glucose, including (**A**) no glucose, (**B**) 30 µg-C/L glucose, (**C**) 90 µg-C/L glucose, and (**D**) 200 µg-C/L glucose. The kinetic constants were obtained from the linearized first-order kinetic modelling. The fitting performances were shown in Figure S10. A *t*-test was used to analyse the significance of differences in BAM biodegradation kinetic constants between groups, “*” means *P* value is 0.01–0.05; “**” means *P* value is 0.001–0.01; “***” means *P* value is 0.0001–0.001; “****” means *P* value is < 0.0001.


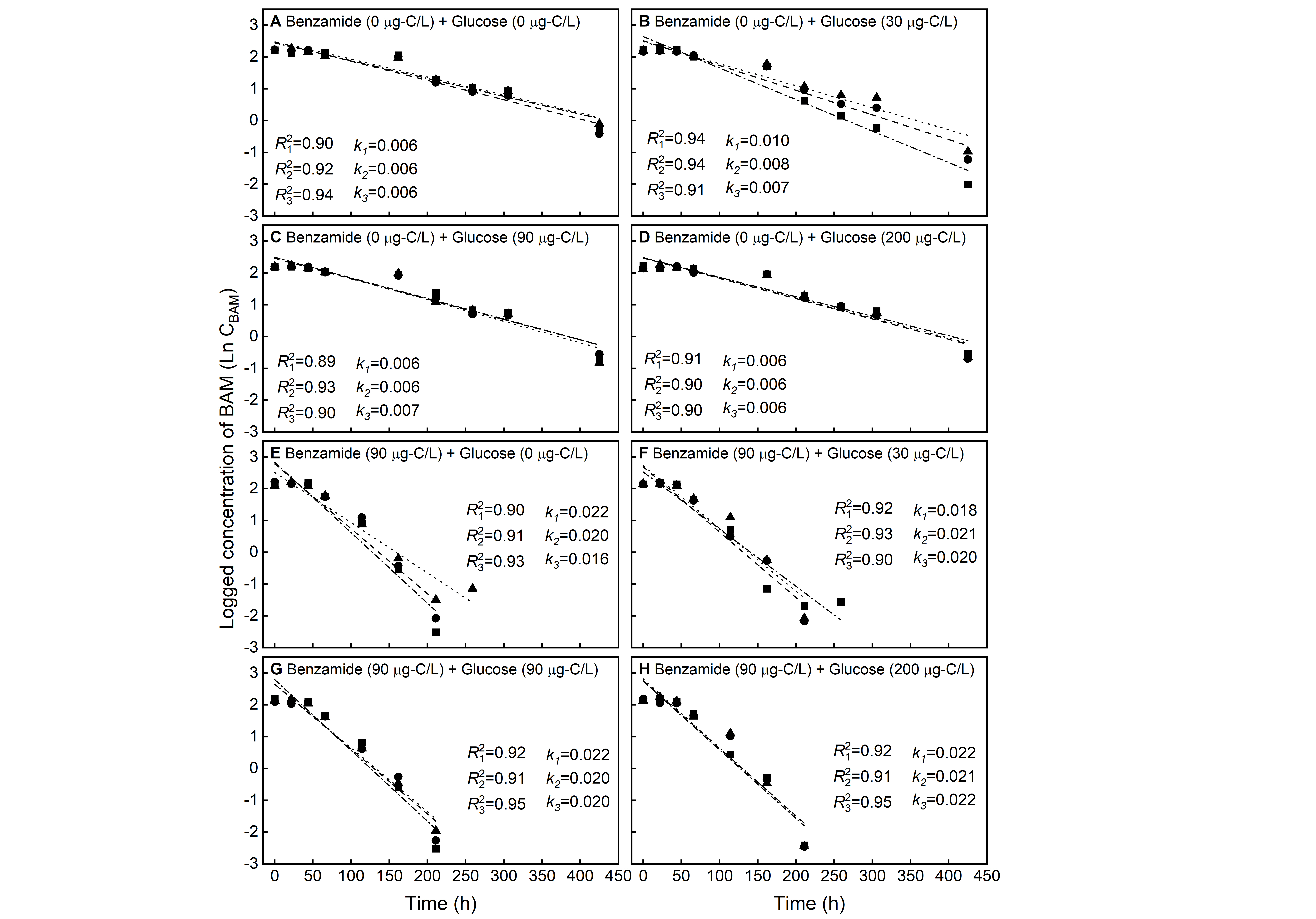


**Figure S10.** Kinetics of BAM biodegradation by MSH1 in the final experiment that explored the effect of auxiliary C-source on growth of MSH1 and concomitant BAM degradation in the presence of SFC-SI. The circle, triangle and square symbols represent the triplicate measured data while the three dot curves show the fitting results obtained from the linearized first-order kinetic modelling. The coefficients of determination (*R*^2^) and kinetic constants (*k*) are shown individually for each triplicate in the figure. It should be noted that this figure only shows the data of experimental groups where MSH1 and SFC-SI were co-inoculated whereas the control groups inoculated with only MSH1 are shown in Figure S12.

**
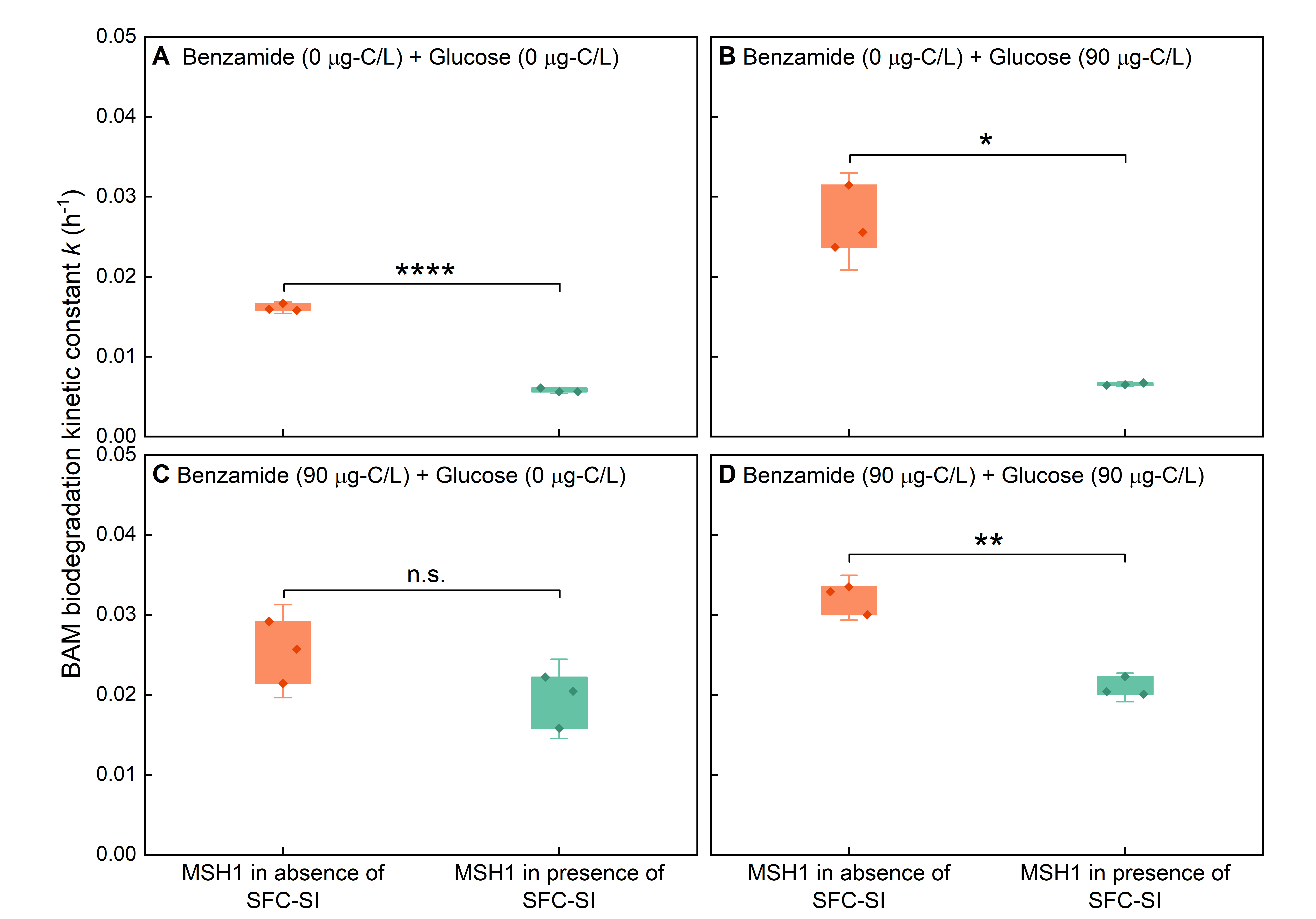
**

**Figure S11.** Statistically significant difference in BAM biodegradation kinetic constants between the condition in absence of SFC-SI and the one in presence of SFC-SI under different concentrations of benzamide and glucose: (**A**) 0 µg-C/L benzamide and 0 µg-C/L glucose, (**B**) 0 µg-C/L benzamide and 90 µg-C/L glucose, (**C**) 90 µg-C/L benzamide and 0 µg-C/L glucose, and (**D**) 90 µg-C/L benzamide and 90 µg-C/L glucose. The kinetic constants were obtained from the linearized first-order kinetic modelling. The fitting performances for the conditions including SFC-SI were shown in Figure S10 whereas the fitting results for the conditions without SFC-SI were shown in Figure S12. A *t*-test was used to analyse the significance of differences in BAM biodegradation kinetic constants between groups, “n.s.” indicates no significant difference with *P* value>0.05, “*” means *P* value is 0.01–0.05; “**” means *P* value is 0.001–0.01; “****” means *P* value is < 0.0001.

**
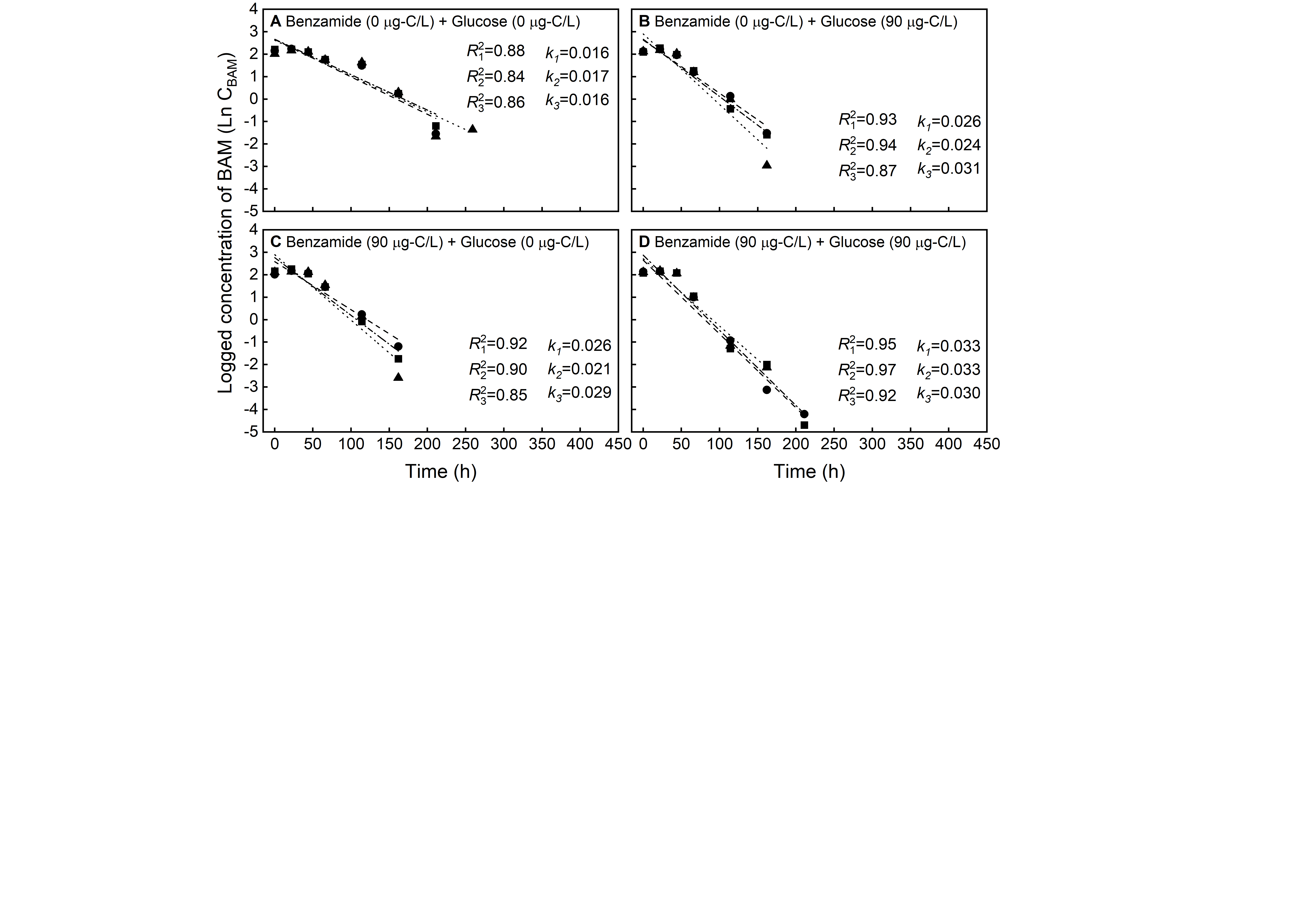
**

**Figure S12.** Kinetics of BAM biodegradation by MSH1 in the final experiment that explored the effect of auxiliary C-source on growth of MSH1 and concomitant BAM degradation in the presence of SFC-SI. The circle, triangle and square symbols represent the triplicate measured data while the three dot curves show the fitting results obtained from the linearized first-order kinetic modelling. The coefficients of determination (*R*^2^) and kinetic constants (*k*) are shown individually for each triplicate in the figure. It should be noted that this figure only shows the control groups where only MSH1 was inoculated whereas the experimental groups inoculated with both MSH1 and SFC-SI are shown in Figure S10.

**
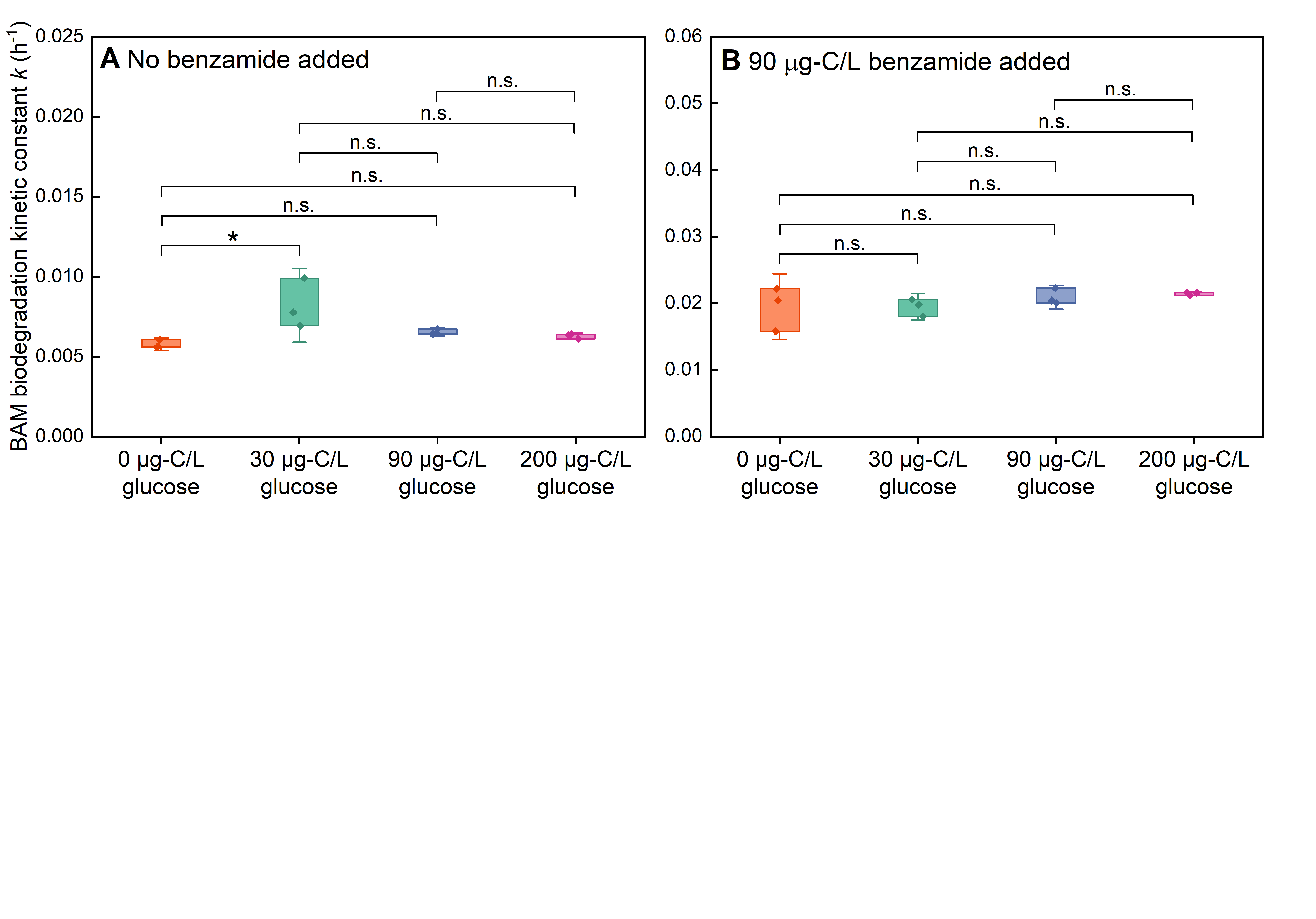
**

**Figure S13.** Statistically significant difference in BAM biodegradation kinetic constants among the conditions with different added amounts of glucose in presence of SFC-SI. (**A**) the comparison under the condition without benzamide, (**B**) the comparison under the condition with 90 µg-C/L benzamide. The kinetic constants were obtained from the linearized first-order kinetic modelling. The fitting performances were shown in Figure S10. One-way ANOVA was conducted to assess whether there were statistically significant differences in means across the groups (*F* value=5.37, *P* value=0.025). Post hoc pairwise comparisons were therefore performed using Tukey’s HSD test. A statistically significant difference was observed only between group without glucose added and group with 30 µg-C/L glucose added in case no benzamide was added (*P* value=0.022, indicated by *). All other comparisons were not significant (n.s., *P* value>0.05).


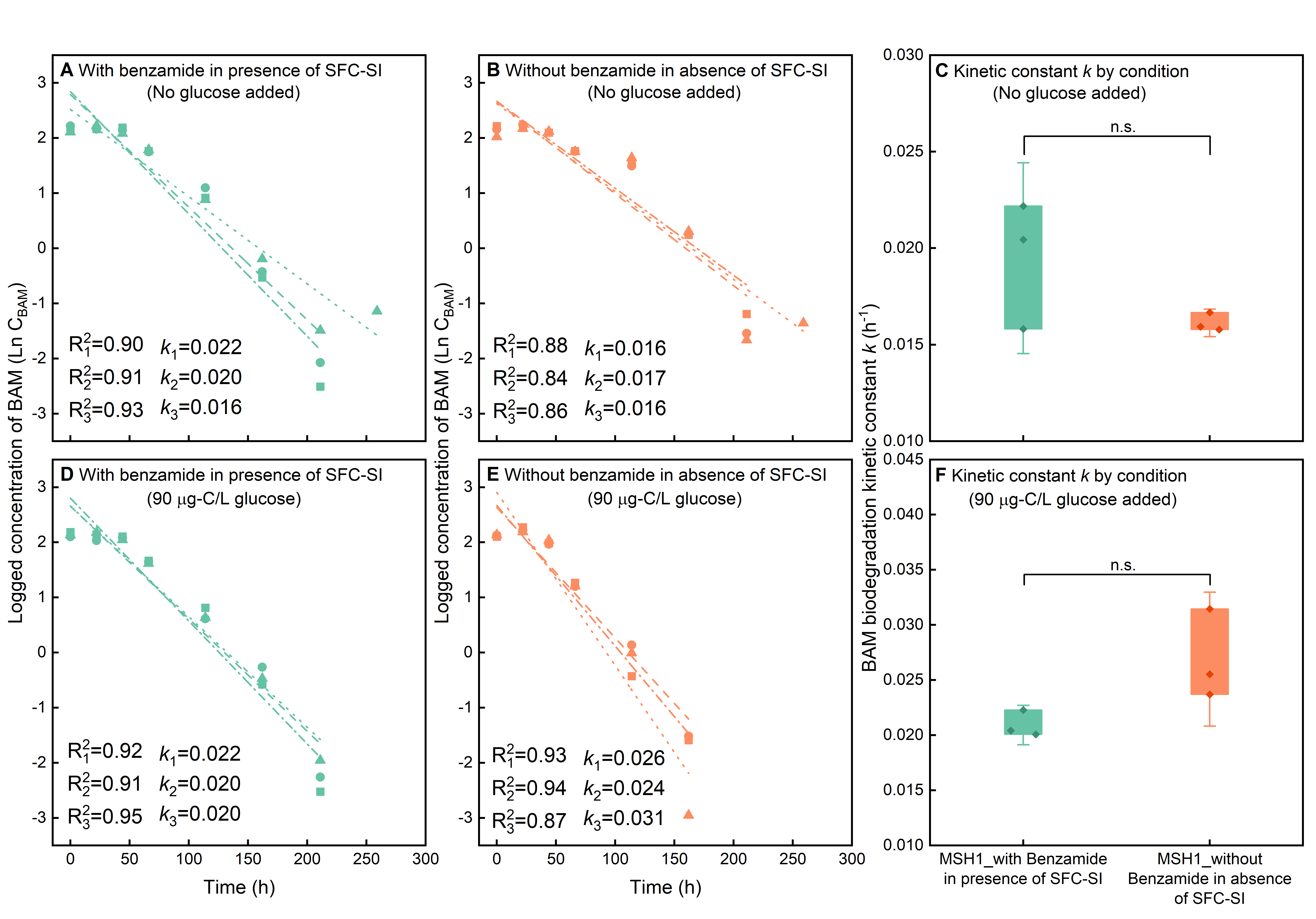


**Figure S14.** Kinetics of BAM biodegradation by MSH1 in the experiment that explored the effect of auxiliary C-source on growth of MSH1 and concomitant BAM degradation in the presence of SFC-SI. The fitting results of kinetic model for the condition (**A**) with benzamide (90 µg-C/L) in presence of SFC-SI without the supplement of glucose, (**B**) without benzamide in absence of SFC-SI without the supplement of glucose, (**D**) with benzamide (90 µg-C/L) in presence of SFC-SI with the supplement of 90 µg-C/L glucose, and (**E**) without benzamide in absence of SFC-SI with the supplement of 90 µg-C/L glucose. The circle, triangle and square symbols represent the triplicate measured data while the three dot curves show the fitting results obtained from the linearized first-order kinetic modelling. The coefficients of determination (*R*^2^) and kinetic constants (*k*) are shown individually for each triplicate in the figure. (**C**) Statistically significant difference in BAM biodegradation kinetic constants under two different conditions without the supplement of glucose. (**F**) Statistically significant difference in BAM biodegradation kinetic constants under two different conditions with the supplement of 90 µg-C/L glucose. A *t*-test was used to analyse the significance of differences in BAM biodegradation kinetic constants between groups, “n.s.” indicates no significant difference with *P* value>0.05.


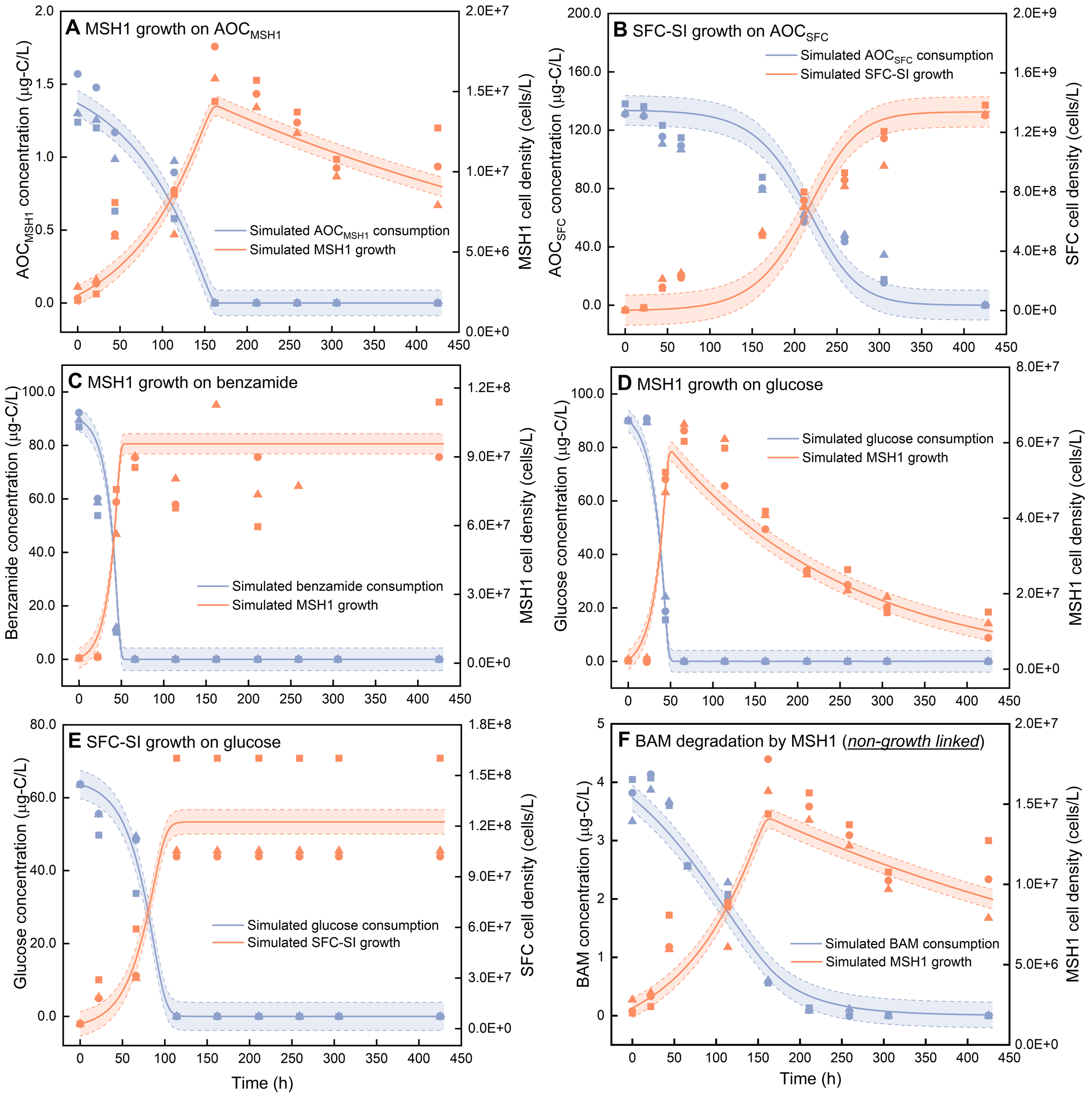


**Figure S15.** Estimation of kinetic parameters from individual configurations based on results from the experiment that explored the effect of auxiliary C-sources on growth of MSH1 and concomitant BAM degradation in the presence of SFC-SI. (**A**) growth kinetics of MSH1 on AOC_MSH1_; (**B**) growth kinetics of SFC-SI on AOC_SFC_; (**C**) growth kinetics of MSH1 on benzamide; (**D**) growth kinetics of MSH1 on glucose; (**E**) growth kinetics of SFC-SI on glucose; (**F**) non-growth linked biodegradation of BAM by MSH1 and MSH1 growth.


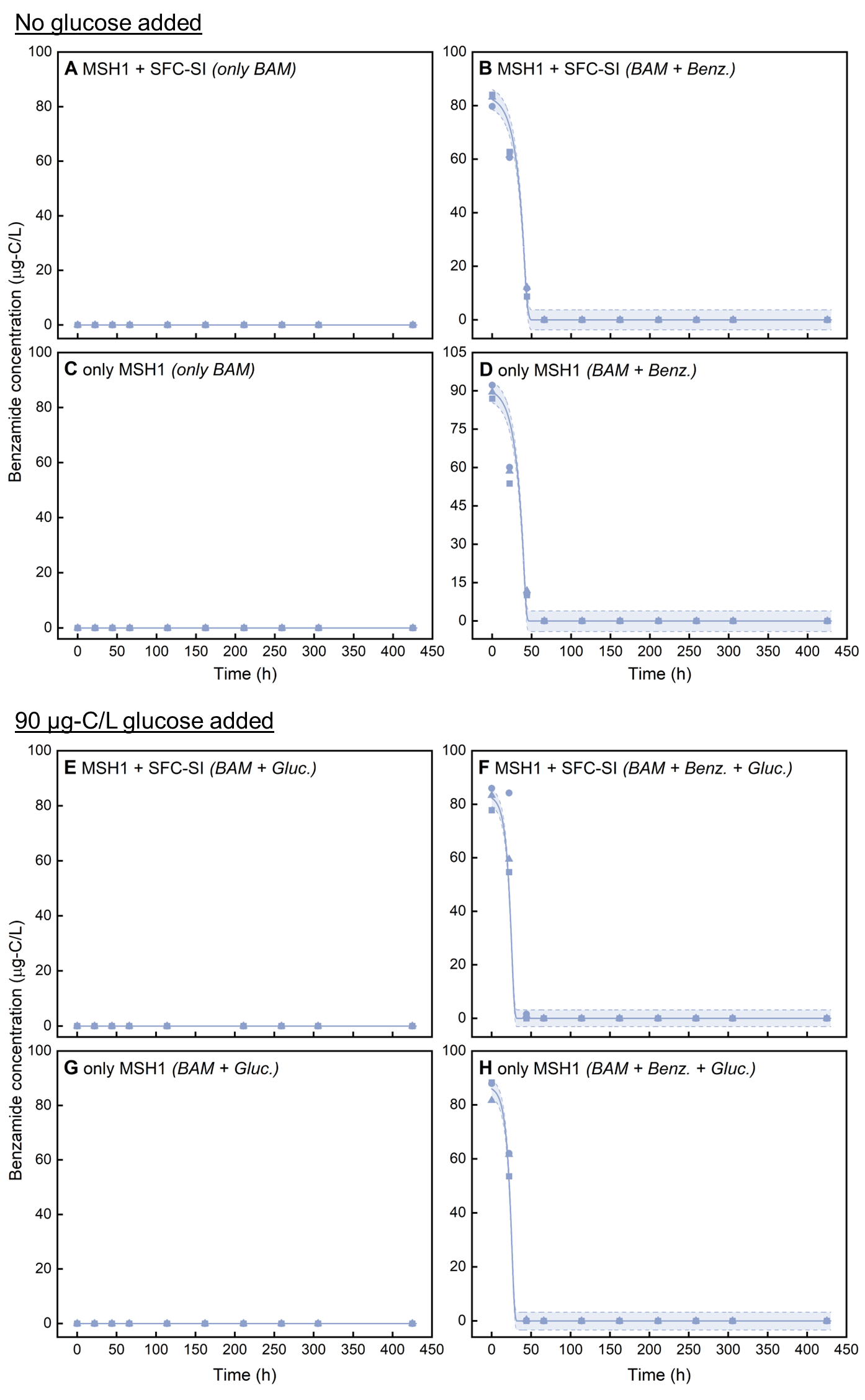


**Figure S16.** Comparison of simulated data (solid lines) by the biokinetic model framework with 95% confidence intervals (shadow areas) with the triplicate measured data (circle, triangle and square symbols) for benzamide biodegradation obtained from different test groups in the experiment that explored the effect of auxiliary C-source on growth of MSH1 and concomitant BAM degradation in the presence of SFC-SI (see Table S1). (**A**) group 1 (MSH1+SFC inoculated, only BAM added), (**B**) group 5 (MSH1+SFC-SI inoculated, both BAM and benzamide added), (**C**) group 9 (only MSH1 inoculated, only BAM added), (**D**) group 11 (only MSH1 inoculated, both BAM and benzamide added), (**E**) group 3 (MSH1+SFC-SI inoculated, both BAM and glucose added), (**F**) group 7 (MSH1+SFC-SI inoculated, BAM, benzamide, and glucose added), (**G**) group10 (only MSH1 inoculated, both BAM and glucose added), (**H**) group 12 (only MSH1 inoculated, BAM, benzamide, and glucose added). The overall results of model evaluation statistics are summarized in Table S5. “Benz.” refers to “benzamide” and “Gluc.” means “glucose”.


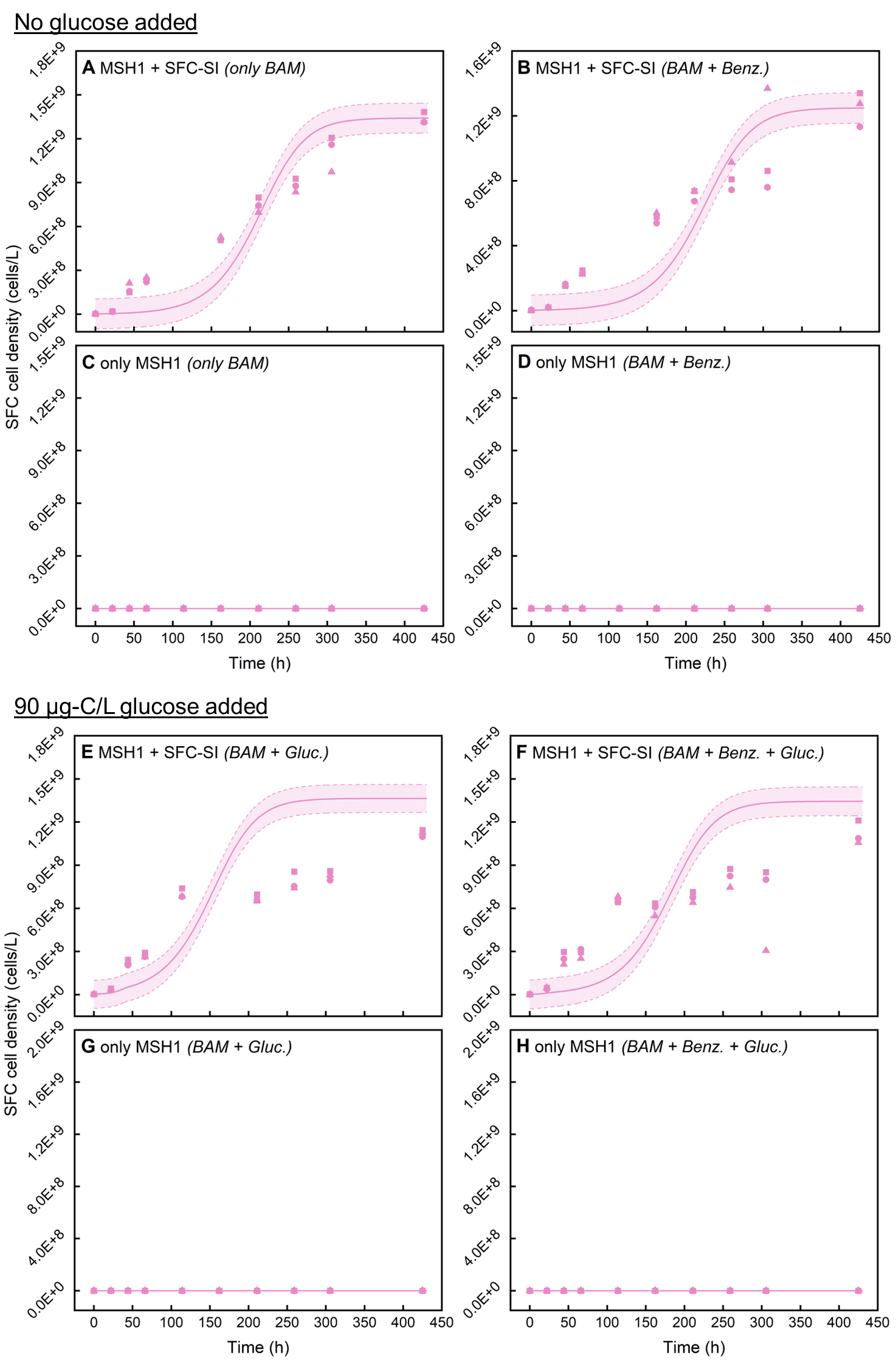


**Figure S17.** Comparison of simulated data (solid lines) by the biokinetic model framework with 95% confidence intervals (shadow areas) with the triplicate measured data (circle, triangle and square symbols) for SFC-SI growth obtained from different test groups in the experiment that explored the effect of auxiliary C-source on growth of MSH1 and concomitant BAM degradation in the presence of SFC-SI (see Table S1). (**A**) group 1 (MSH1+SFC inoculated, only BAM added), (**B**) group 5 (MSH1+SFC-SI inoculated, both BAM and benzamide added), (**C**) group 9 (only MSH1 inoculated, only BAM added), (**D**) group 11 (only MSH1 inoculated, both BAM and benzamide added), (**E**) group 3 (MSH1+SFC-SI inoculated, both BAM and glucose added), (**F**) group 7 (MSH1+SFC-SI inoculated, BAM, benzamide, and glucose added), (**G**) group10 (only MSH1 inoculated, both BAM and glucose added), (**H**) group 12 (only MSH1 inoculated, BAM, benzamide, and glucose added). The overall results of model evaluation statistics are summarized in Table S5. “Benz.” refers to “benzamide” and “Gluc.” means “glucose”.

**Document S4**

**References**

(1) Sekhar, A.; Horemans, B.; Aamand, J.; Sorensen, S. R.; Vanhaecke, L.; Vanden Bussche, J.; Hofkens, J.; Springael, D. Surface colonization and activity of the 2, 6-dichlorobenzamide (BAM) degrading *Aminobacter* sp. strain MSH1 at macro- and micropollutant BAM concentrations. *Environ. Sci. Technol.* **2016**, *50* (18), 10123–10133.

(2) Schlechter, R. O.; Jun, H.; Bernach, M.; Oso, S.; Boyd, E.; Muñoz-Lintz, D. A.; Dobson, R. C. J.; Remus, D. M.; Remus-Emsermann, M. N. P. Chromatic bacteria – a broad host-range plasmid and chromosomal insertion toolbox for fluorescent protein expression in bacteria. *Front. Microbiol.* **2018**, *9*, 3052.

(3) Raes, B.; Wang, J.; Horemans, B.; Dirckx, L.; Waldherr, S.; Kohler, H.-P. E.; Springael, D. The growth yield of *Aminobacter niigataensis* MSH1 on the micropollutant 2,6-dichlorobenzamide decreases substantially at trace substrate concentrations. *Environ. Sci. Technol.* **2024**, *58* (6), 2859–2869.

(4) Rhine, E. D.; Mulvaney, R. L.; Pratt, E. J.; Sims, G. K. Improving the berthelot reaction for determining ammonium in soil extracts and water. *Soil Sci. Soc. Am. J.* **1998**, *62* (2), 473.

(5) Narayana, B.; Sunil, K. A spectrophotometric method for the determination of nitrite and nitrate. *Eurasian Journal of Analytical Chemistry, Moment Publications*. **2006**, *4*, 204–214.

(6) Holman, W. I. A New technique for the determination of phosphorus by the molybdenum blue method. *Biochem. J.* **1943**, *37* (2), 256–259.

(7) Hammes, F.; Egli, T. A Flow Cytometric Method for AOC Determination. *Techneau* **2007**, 1-20. Report No.: 3.3.1.

(8) Liu, L.; Helbling, D. E.; Kohler, H. P. E.; Smets, B. F. A model framework to describe growth-linked biodegradation of trace-level pollutants in the presence of coincidental carbon substrates and microbes. *Environ. Sci. Technol.* **2014**, *48* (22), 13358–13366.
